# Supplementary material for: Notch coordinates self-organization of germ layers and axial polarity in sea anemone gastruloids
Source: Nat Commun. 2026 Jun 19;17:6182. doi: 10.1038/s41467-026-74441-x (PMC13370024; doi:10.1038/s41467-026-74441-x)
Supplement: Supplementary file 1 — Supplementary Information [file 41467_2026_74441_MOESM1_ESM.pdf]

Supplementary material

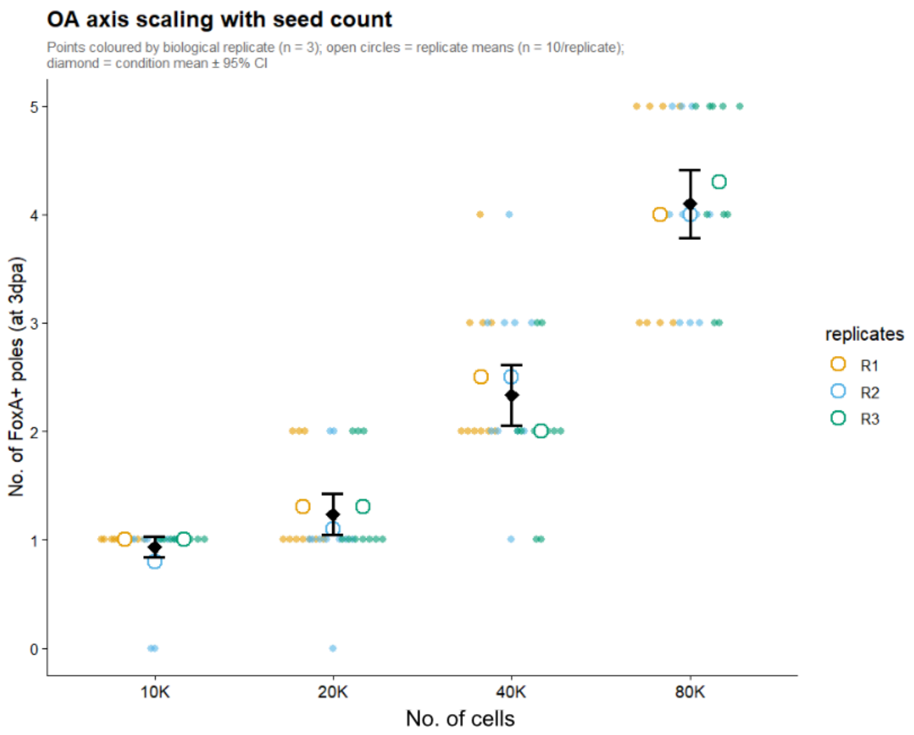

Figure S1. Scaling of axes with increasing seed counts measured by no. of FoxA+ poles at 3dpa.

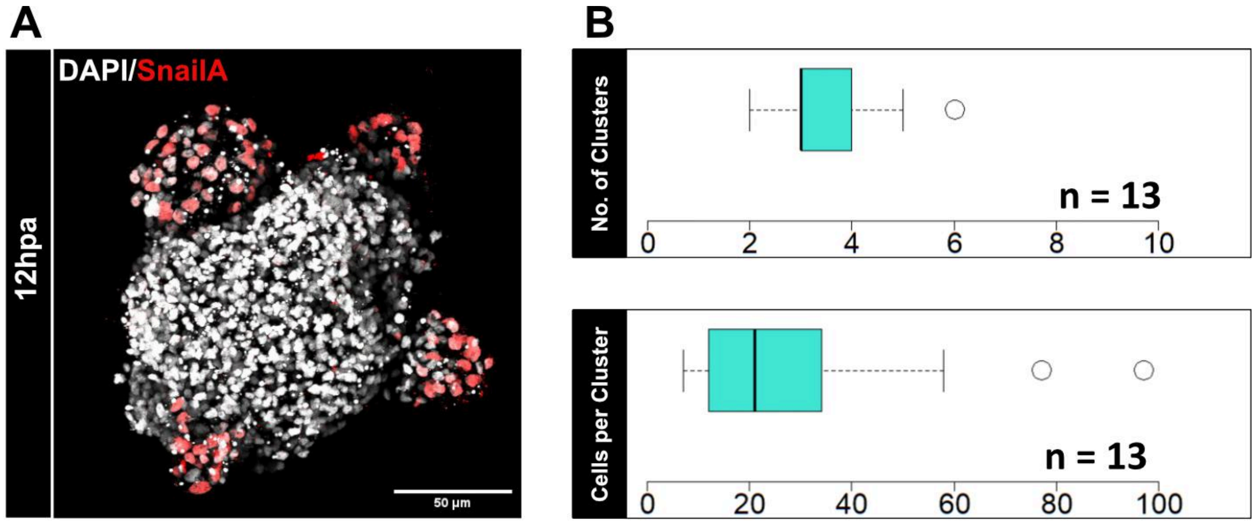

Figure S2. Quantitative analysis of mesodermal cluster metrics just prior to ingression.

(A) Antibody staining for SnailA demarcating peripheral mesodermal clusters at 12hpa. DAPI staining marks the nuclei (Scale bar: 50μm)

(B) Cluster metrics quantified from images show on average 3 clusters per gastruloid and 20 cells per cluster per gastruloid. ‘n’ represents the number of gastruloids.

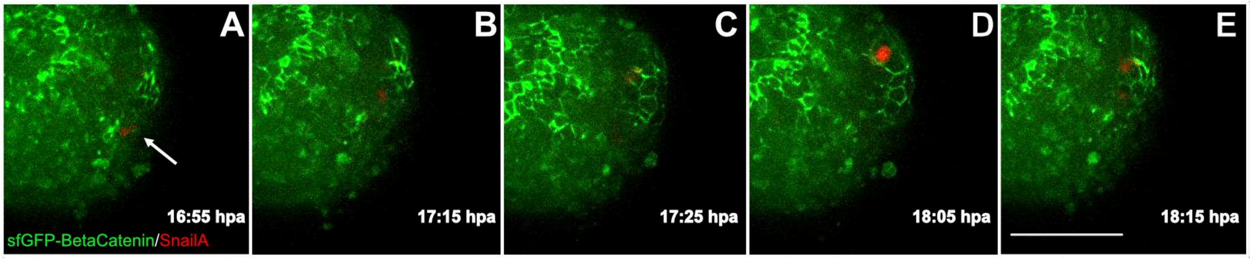

Figure S3. SnailA+ cells ingress at regions of non-epithelialization.

(A-E) Time Lapse images of transgenic SnailA+ cells sorting in gastruloids with cell junctions marked by sfGFP-BetaCatenin. White arrow marks cell ingression at point of non-epithelialization. (Scale bar: 100μm)

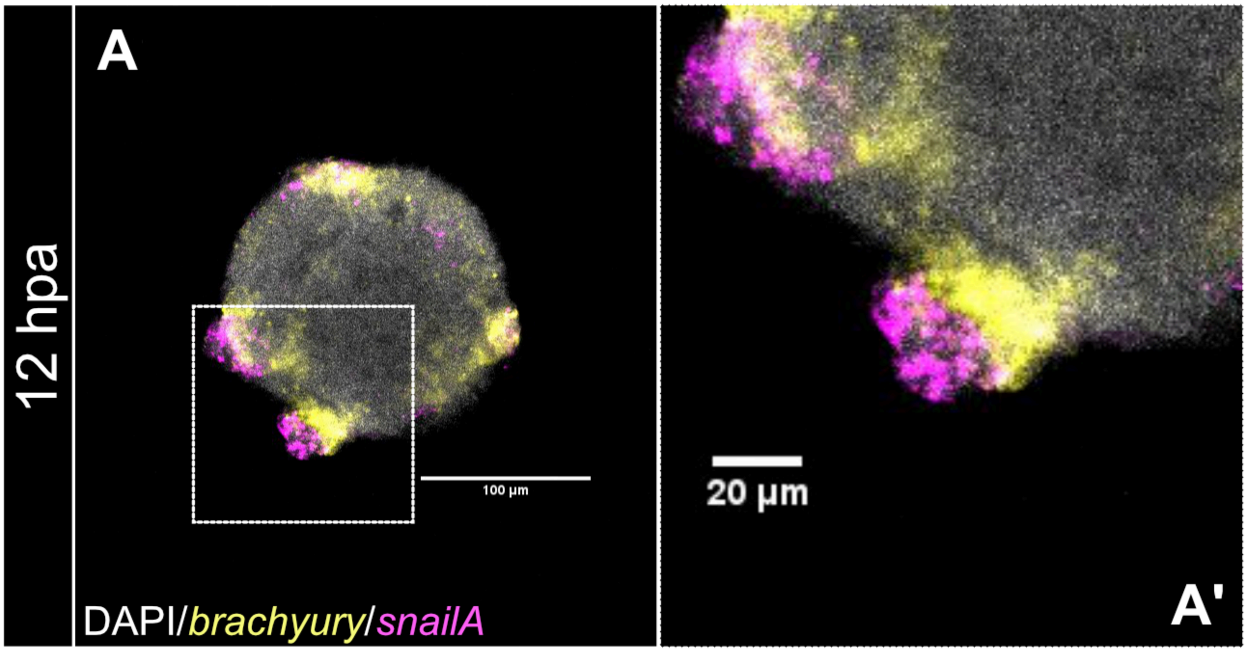

Figure S4. *Brachyury*+ cells form a clear boundary and exhibit no intercalation within the mesoderm.

(A-A') dFISH analysis of *Brachyury* and *SnailA* (Mesoderm) in 12hpa gastruloids (Scale bar: 100μm). (A') closeup showing clear boundary between both domains (Scale bar: 20μm)

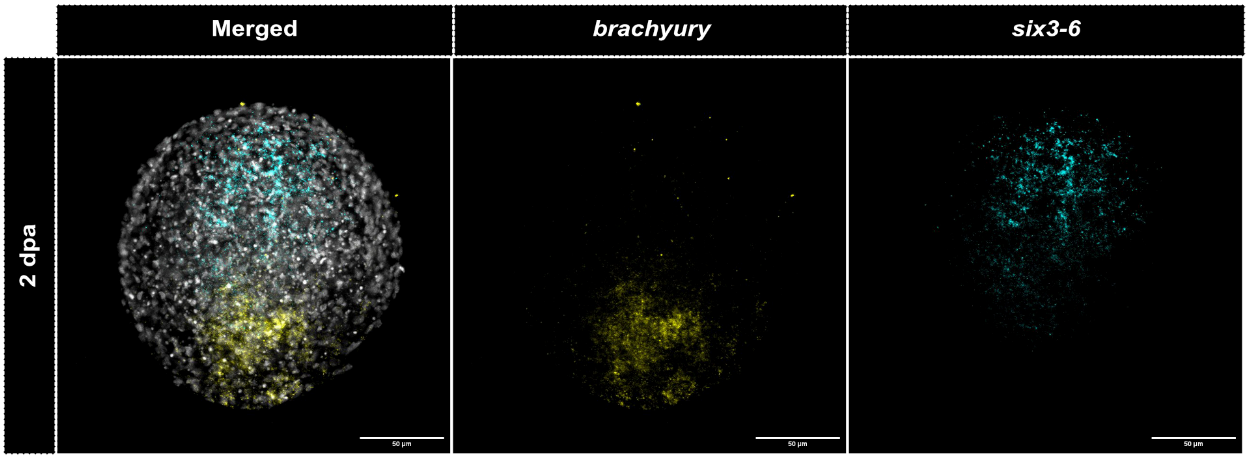

Figure S5. Hybridization Chain Reaction staining of *brachyury* (oral marker) and *six3-6* (aboral marker) in 2dpa gastruloids (Scale bar: 50µm).

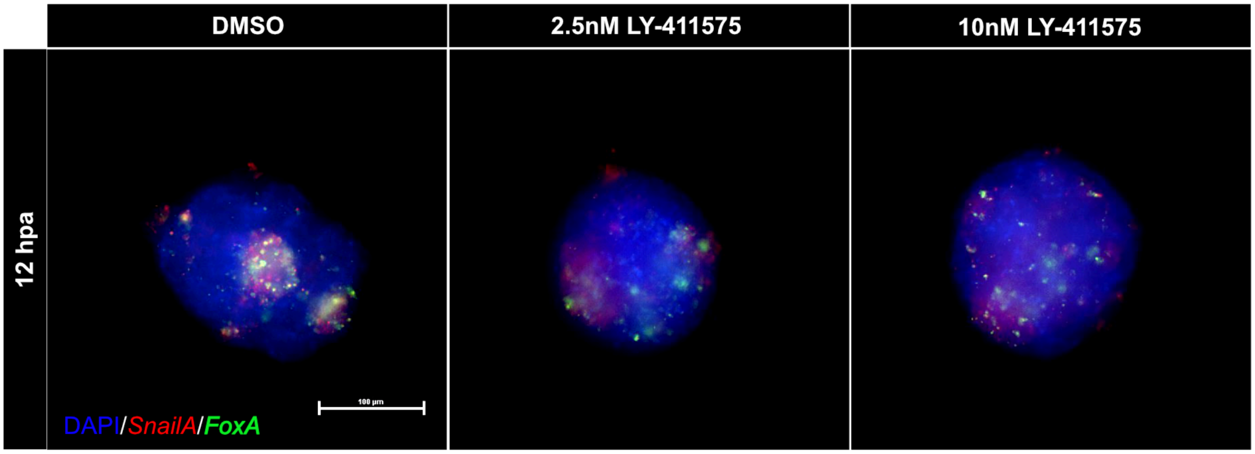

Figure S6. Notch signaling maintains endo-mesodermal boundary in gastruloids.
dFISH analysis of endoderm (*FoxA*+) and mesoderm (*SnailA*+) boundary formation in notch signaling inhibited gastruloids at 12hpa (Scale bar: 100µm).

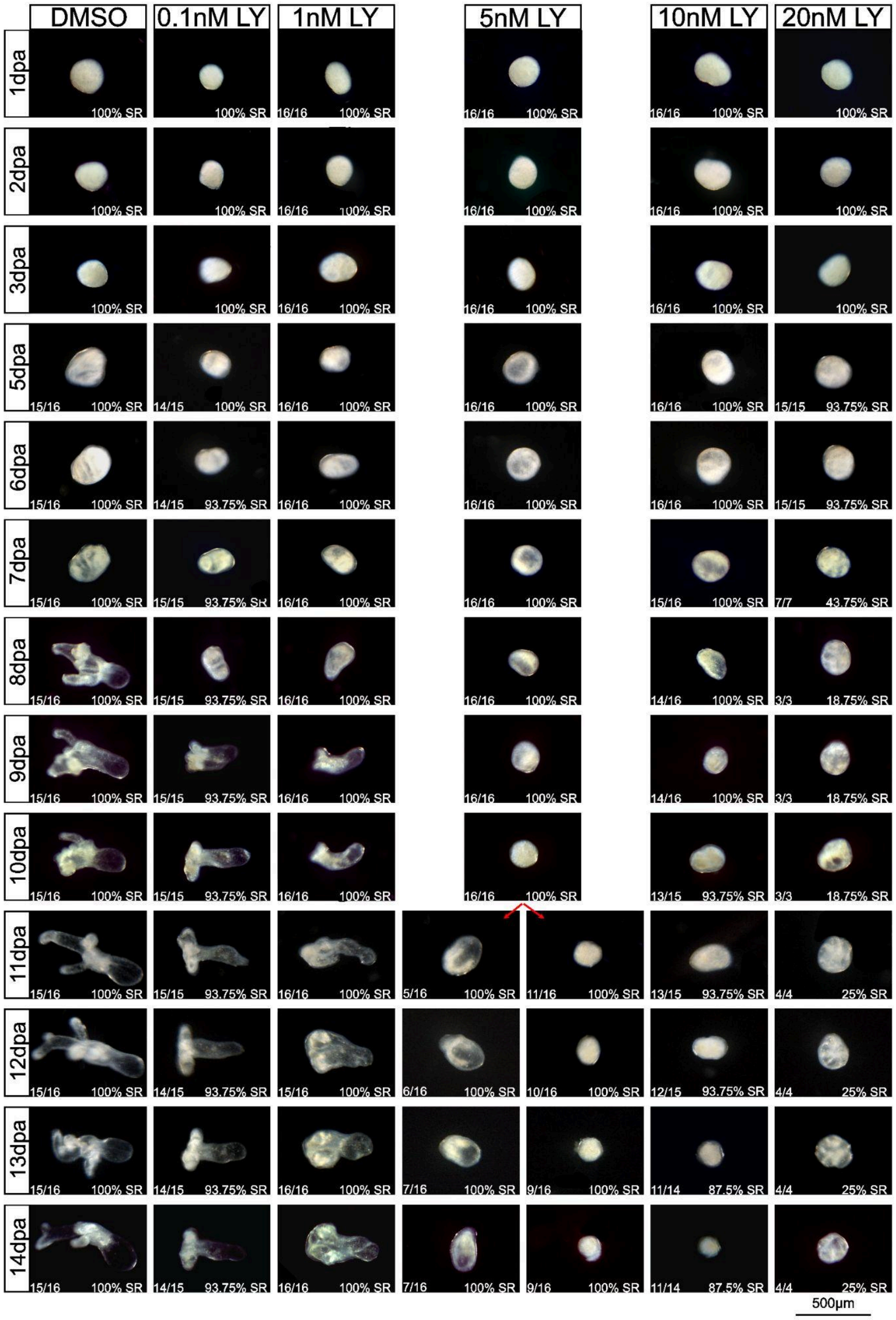

Figure S7. Notch signaling is essential for gastruloid head formation.

Dose dependent loss of gastruloid head structures (tentacles and pharyngeal tissue) with LY-411575 treatment until 14dpa. Red arrows in the 5nM LY-411575 treatment column represent a split into dual phenotypes. Fraction on the bottom left of each picture represents no. of gastruloids with a particular phenotype out of total. dpa;days post aggregation.SR;Survival rate (Scale bar: 500μm)

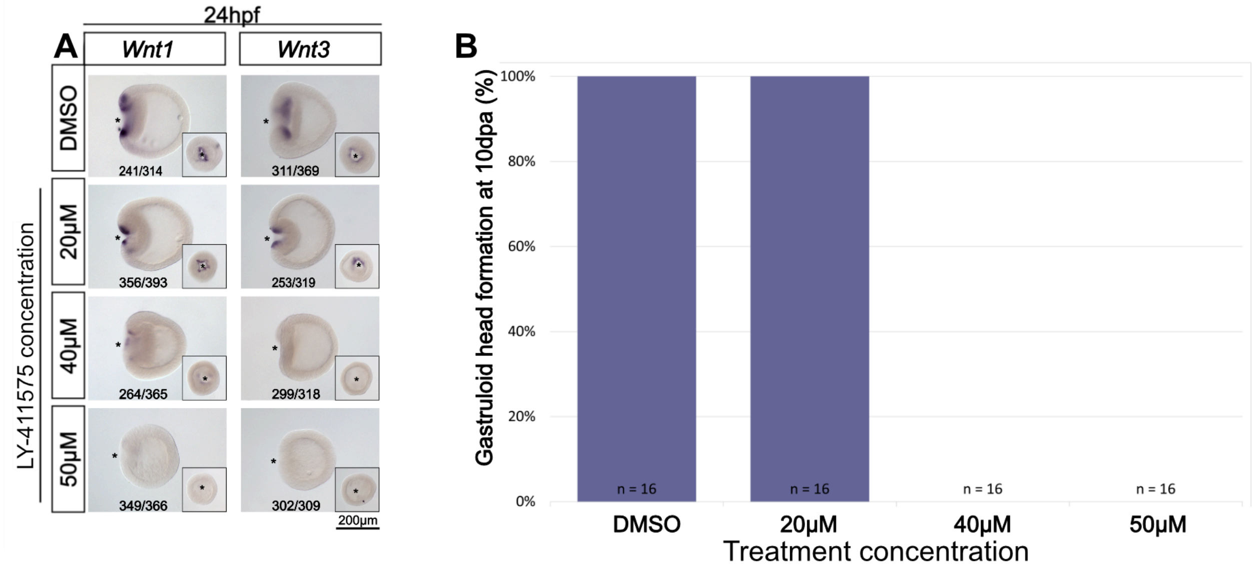

Figure S8. Pre-existing endoderm is essential for axis reestablishment.

(A) Gradual ablation of axial organizers Wnt1 and Wnt3 at 24hpf by different concentrations of Notch signaling inhibitor LY-411575. Ablation was visualized by *in situ* hybridization. (Scale bar: 200µm)

(B) Effect of different levels of pre-existing organizers at 24hpf on gastruloid head formation at 10dpa.

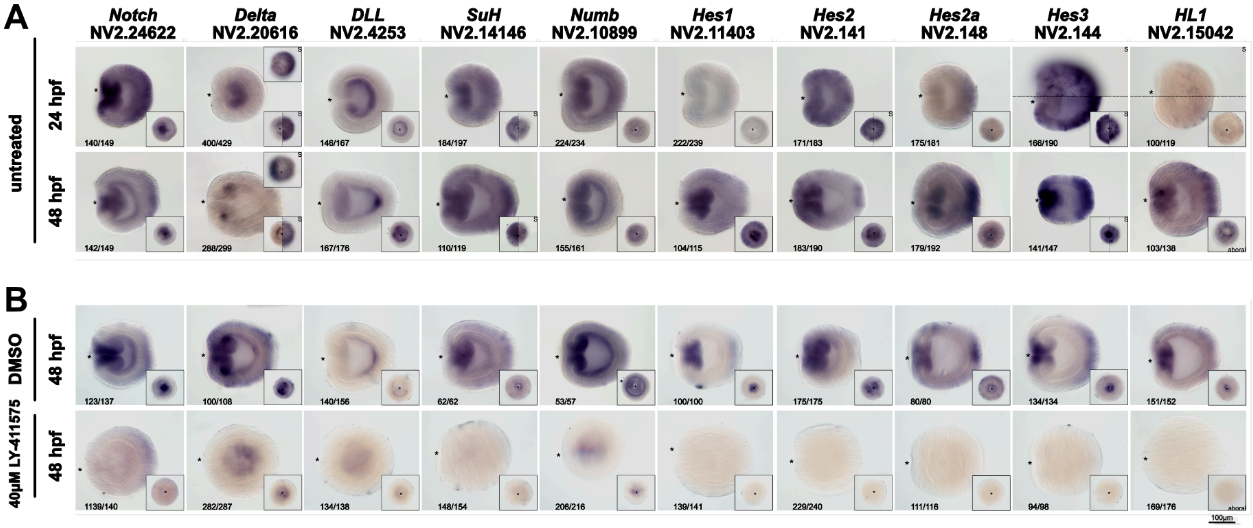

Figure S9. Characterization of canonical Notch signaling components.

(A) Characterization of annotated Notch signaling components by *in situ* hybridization in 24 and 48 hpf wild type embryos. S represents the surface view of the embryo.

(B) *In situ* hybridization expression analysis of Notch signaling components in 48hpf embryos treated with DMSO and LY-411575 from 24hpf to 48hpf. Asterisk represents the oral pole (scale bar: 100µm)

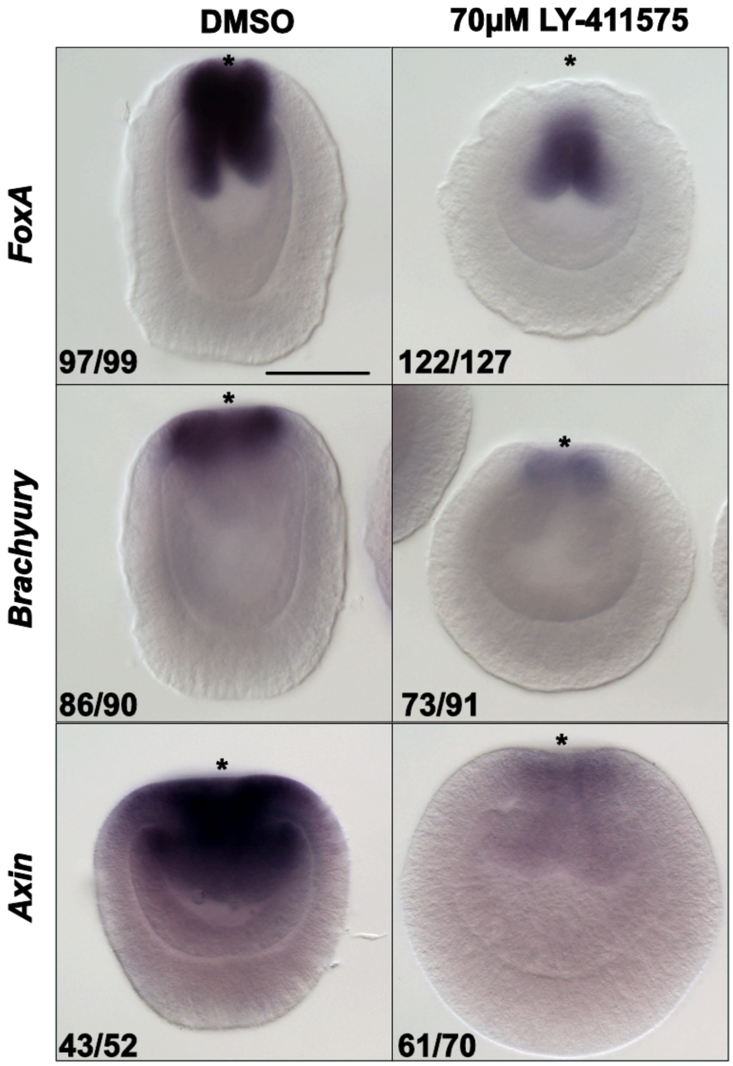

Figure S10. Notch signaling maintains endoderm identity post-gastrulation.

In situ hybridization of endodermal genes *foxA*/*brachyury*, and BetaCatenin target *Axin* in 48hpf embryos treated with DMSO and LY-411575 from 24hpf to 48hpf. Asterisks denote the oral pole. (Scale bar: 100μm)

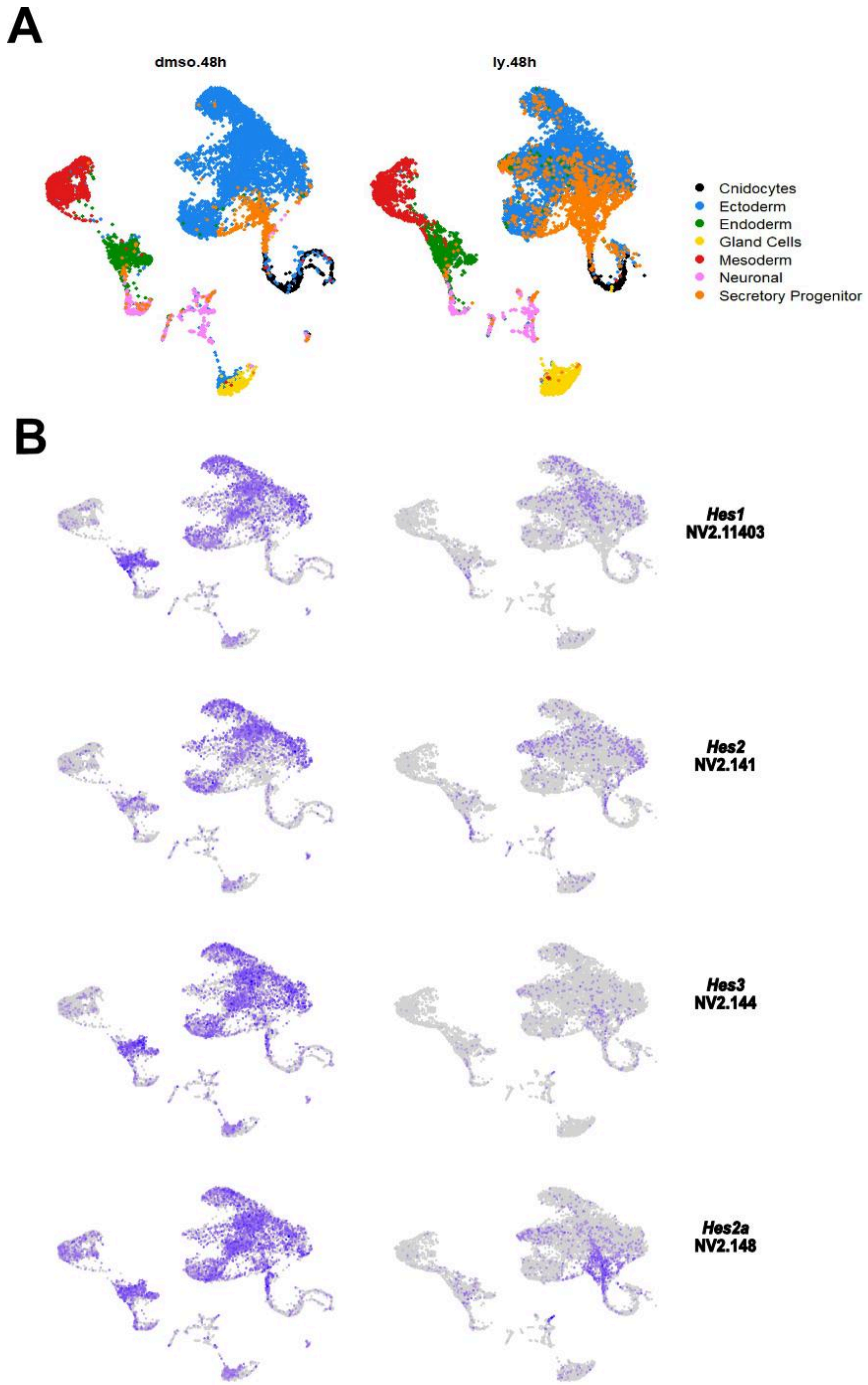

Figure S11. Knockdown of Notch signaling targets in LY-411575 treated dataset.

(A) UMAP dimensional reduction showing annotated clusters in libraries capturing response of embryos to Notch signaling inhibition, treated from 24 to 48hpf.

(B) Feature plot depicting knocked down expression of previously reported putative targets of Notch signaling in LY-411575 treated embryos compared to DMSO treated control embryos.

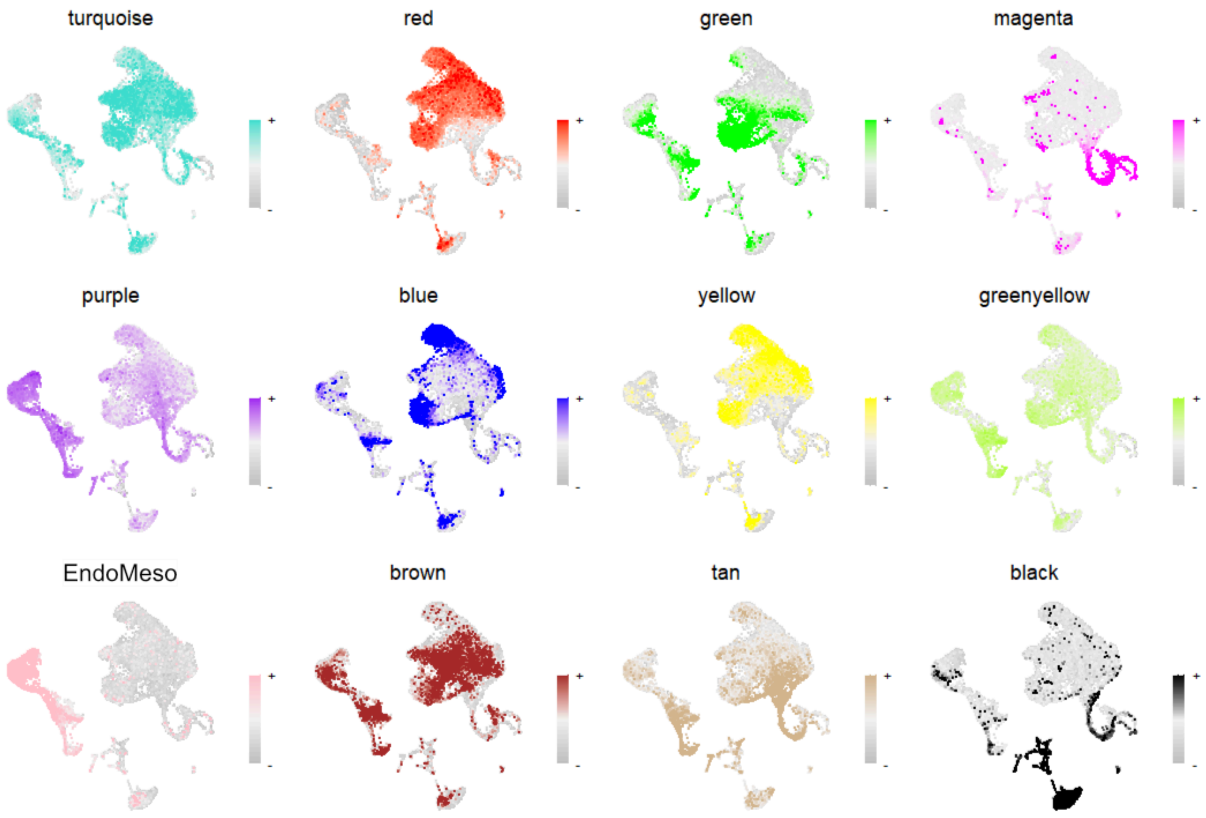

Figure S12. WGCNA gene module analysis.

Feature plot showing eigen gene expression of identified gene modules.

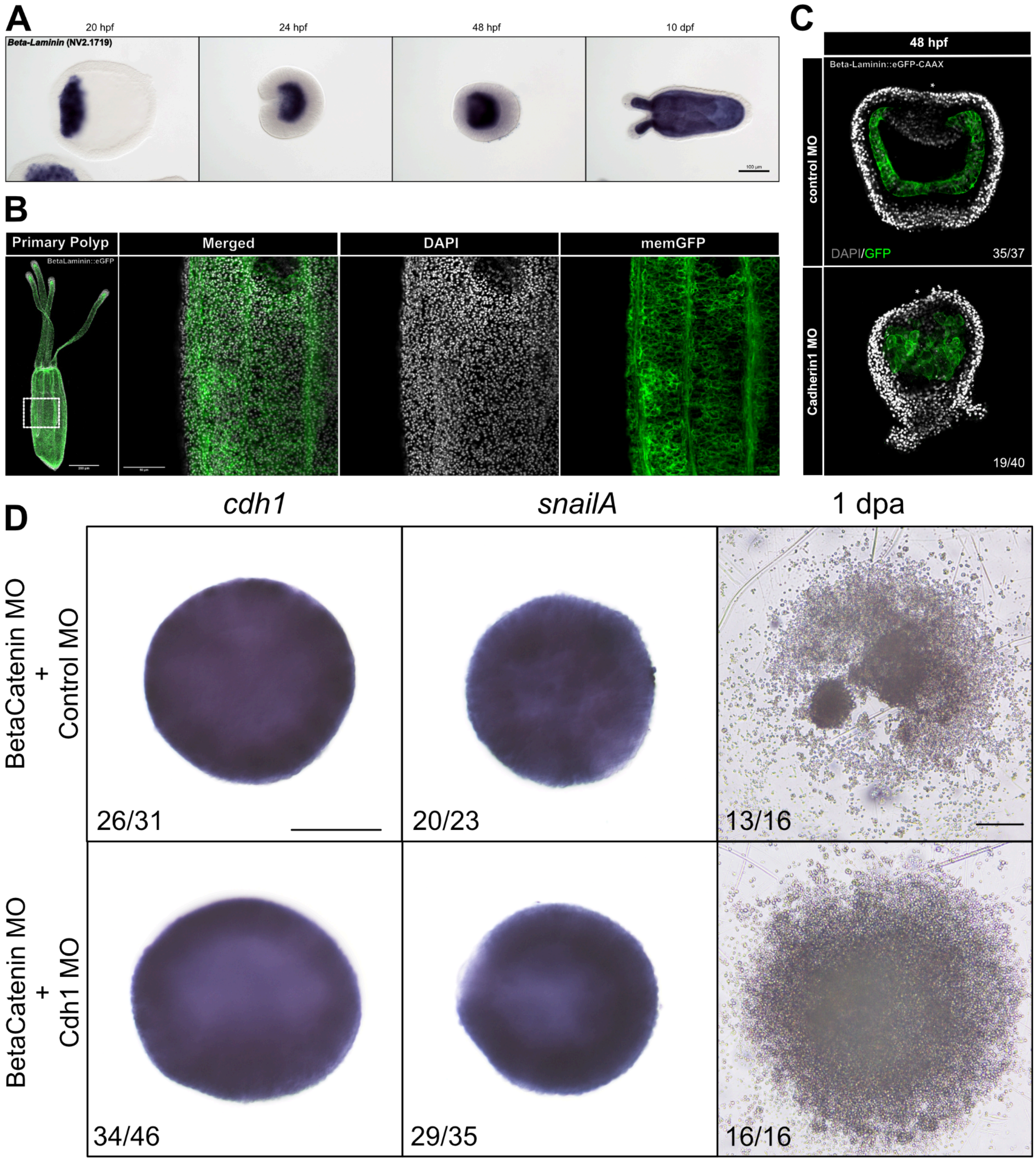

Figure S13. Mesodermal adhesion is mediated by Cadherin1

(A) Characterizing expression of β-*Laminin* in development by *in situ* hybridization (Scale bar: 100μm).

(B) F1 generation primary polyp of β-*Laminin::eGFP-CAAX* line displaying membrane fluorescence in mesoderm.

(C) Anti-GFP antibody staining of β-*Laminin::eGFP-CAAX* line embryos post knockdown by control and cadherin1 morpholino at 48hpf.

(D) First 2 columns denote expression of mesodermal markers *cadherin1* and *snailA* in embryos co-injected with **β**-catenin MO/control MO (row1), and **β**-catenin MO/cadherin1 MO (row2). Final column represents aggregates made with the respective injected embryos 1dpa (Scale bar: 100µm).

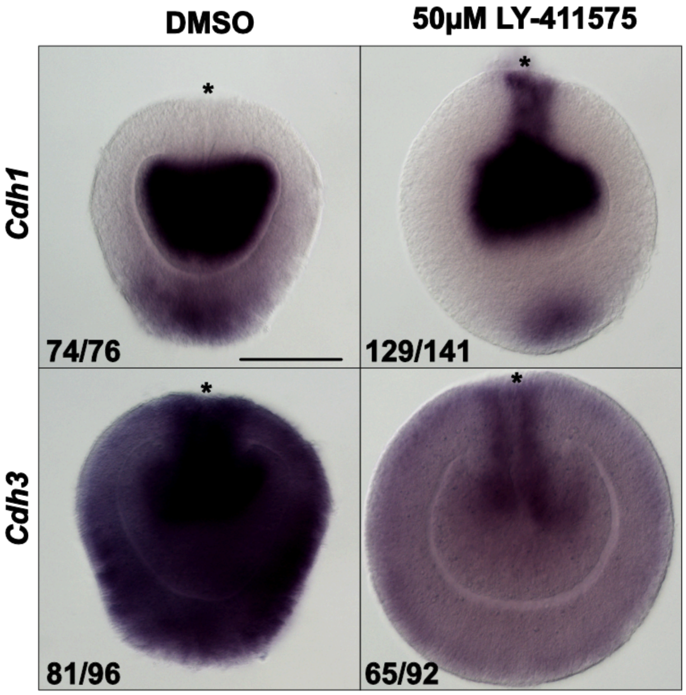

Figure S14. Notch signaling maintains Cadherin Switch.
In situ hybridization of *Cadherin1* and *Cadherin3* in 48hpf embryos treated with DMSO and LY-411575 from 24hpf to 48hpf. Asterisks denote the oral pole. (Scale bar: 100µm)

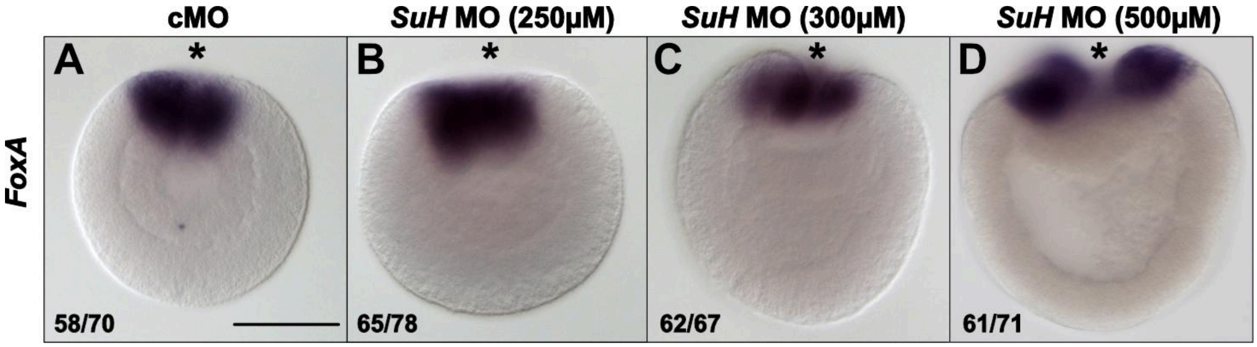

Figure S15. Low dose *SuH* MO injected embryos display no gastrulation delay.
(A-B) Low concentration of *SuH* MO (B) injected embryos exhibit no phenotype and is comparable to the control morpholino (A).
(C-D) Higher concentrations of *SuH* MO causes delay in gastrulation.
Asterisks denote the oral pole. (Scale bar: 100µm)

Common Name	Gene ID	Primers	Sequences
Notch	NV2.24622	F	AGCTGAGAAGATTTGGTTCATTG
		R	TTGACCAGTCTGATAATAACTCCA
Delta	NV2.20616	F	CAATCGGTACACCTGCTC
		R	CAAGTTCCATCACCAAATGC
DLL	NV2.4253	F	GTCTTTTGGAGTCGCTGTGG
		R	GCTCCCGTTAGAATCACACG
SuH	NV2.14146	F	AAGATTTCACTTCGACAGCT
		R	GGACCCATACCCTCATAGAA
Numb	NV2.10899	F	TCTGACTGCATTCGTGTCG
		R	TGCTAGAGAGGCATTTGACTG
Hes1 (HEY)	NV2.11403	F	CGACGAAAGCCAACCGGA
		R	CAAATTCTGCAATCACCACG
Hes2	NV2.141	F	CGACGAAAGCCAACCGGA
		R	CAAATTCTGCAATCACCACGG
Hes2a	NV2.148	F	AACTAACTACAACGCTAGCG
		R	TTCAAATTGTCCTCCCCATT
Hes3	NV2.144	F	TCAACACAAACTCTCAACA
		R	TCGAGGTATTAACTTTTATTGCT
HL1	NV2.15042	F	CCAAGCTGGAAAAAGCGGAC
		R	GTTCTCTGCTTGGGAAGCCT
FoxA	NV2.11441	F	ATGATGGAGCACACGGG
		R	CGAAAGATTTGATATACCACAACTC
Brachyury	NV2.10624	F	ATGCACTCGGACGAGAAGAAAC
		R	TTAAGCTTGCGGTATGGTGTTCC
Axin	NV2.20395		nucleotides 1–1123 of Genbank JQ959548
Cdh1	NV2.4715		Pukhlyova et al. 2018
Cdh3	NV2.4928		Pukhlyova et al. 2018
SnailA	NV2.472	F	ATGCCCCGCTCGTTTCTAG
		R	CTATCCTTGTGACGGGCA
Wnt1	NV2.12225	F	ATGCAACGATTCAGCGCAGCGATC
		R	TTATAAGCAGTTACTAATGATCC
Wnt3	NV2.12463	F	ATGAGAGTAATTATCACTGCGATTG
		R	TTATTTACAAGTGTAGATGTTAACCACTT

Table S1. List of primers for in situ probe synthesis

**FoxA (5‘ to 3‘)**
AGGAGGTAATCTGCACAAGTCGCTAGCGGAGACACAGTGAACACCAACACCAGCAGCGCGCAACGTCAACCGGCAAACTGGATCCTCAGTACA GAAACCTCGAGATAGAGATGATGGAGCACACGGGGGTGCCTCCAGCGGCCATGCAAGACCCATCACAAAACCCGCACGAGCTCAAGAAATCCA AGGACAAGGAGAAAGCGTATCGCCGGAGCTACACGCACGCCAAGCCGCCATATTCATATATCTCACTCATCACGATGGCCATTCAACAGAGCCCA AACAAGATGCTCACACTGAGCGAGATCTACCAATTCATCATGGACTTGTTTCCCTACTACAGGCAAAACCAACAGCGCTGGCAGAACTCTATCCG GCACAGTTTATCATTCAATGATTGCTTCGTGAAGGTGCCGCGCTCTCCTGACCGCCCCGGGAAAGGCAGTTACTGGACTCTCCACCCGGACTGC GGTAATATGTTCGAGAACGGGTGCTACCTTCGCAGGCAGAAGCGCTTCAAAGCCGAGAAAAAACCGGACCTGAGTCACCTTAGCAAGGTGAGC AGTATGACACACAACCCGGTCACAGTACAAAGCATGGCGAAGAGCATGGCGGCACAACCTCGCTCAATGGGAACCCCTAGCTTCCTTGCACCG TCTCCGTACGGGACGGCTATGGGCATGGGACACGTTGGGAGCATGACCGCCATGGGTATGGCAGGTATGCCCATGAATAAGTCGTTTAATCACC CATTCGCTATCAAGAATATCATCGCGCAAGATCACGAAGCTGAGCTTCGAGGCTACGACCCCATGCACTTCAGTCCTTATCATCCATCACTCCAAT CCATGGGTTCCCTAGGACTCCCTAAATCCGCCTACGAATCGCAACCTATCACGACGGATACGAGTCCGTACTACCAGGGTTGCGTCTTCACGCC GTCGAGTTGTGGTATATCAAATCTTTCGTGAACTTAGCGCAGCACTTAGAACACTAGAAGTAGAAATTATTCTAAGAAAAGCTGAATATTATGATAAA CCTGTATATATAAACATTGAGGCACTGAAAATTGAGCTATCCGACGAATATCTCGTCTATGTACAATGTGCTTGAGCTTTCGTATGATTATATTGATCA TTTTTTTACTACAAGCACAACTAGATTTCGTAAAGAGCTAGAAAATTTAATAATTTTATGAACAATCAAAAATGTAAAATAGAAGATGAAAATGTTAGG CTAGGAAAAACAAAAATGATTAGAGTATTTTGAAGAAAGCAAATCTTCCGAAATCAAAATATTTTGAAATTGAATTAACAGACACAAGTGATTATTAG CTAAGAAAATGTTTGTAAAGATTCCTATATACACATATATATTGTTTCGCAAACTTGTTCTGTTGAAGCTGACCATTAGATGAAAGAGCGTGCAGTTA ATATTTGGCTTTTCGACAGTCGCTTTCTGTATTCGGGGCATAACATTGACTGTTGCTCAGTTTGTTCTCGTGTCAACTCTGTATTTATCATTGCTTCA CTTTTATTTATACTAGTCAGGCGAGGGAACGGTTTTTATTGTAAATGTACTCTCATGGTCAACGTTTGTGGATACTTGAAGAAAATAAATTTTTAACG AATC
**Cdh1 (5‘ to 3‘)**
GGCTGTTATCATGATTATTTTTAGTGAAAGTCACCGCGAGAACTAACGCTTTCTGGCTGATGTTGGCATGACCTTTGCAGTAATCTAGACGCCATT CAAACGATATCCCAGGCTTAATCTACATTTCCGACCAATCCGAAGCCAGCGATCAAGGACTTTATAAATTCTCAAGCGAGTTATTTTTGCGTATCAA GTTAGGGAGTCTTCGGTTCAACCTTGTAAGGCGCTCGTTCAACGGCCATATGCTATGGGTTTTTAAACTCACGGGTTCCTAATAACTAGCCACAAA ATGAGTGCTGGCCGGCTGGCGGCTGTGCTAACCACTCCACTATTATTTCTTAGTTTGCTTAAAACTTTTCAACTGGCAAAAGCGCAGGACACTTT GATCGAAGTGAATTTCGATGAGGGTCGACCGGCTCGTTCTTCTGTCTACTTGTTCGACTCTTCTAGTGGCGATGTATTCTCGCTGTACCAAGCAG ATCCGACAGTGCCGCTGCTATTTCAAATATCTGAGGTTGGACACGTAACATCGACTCAAGAAATCGAGTACGAAATAGGAAAAACAAATAAATACG ACTTGACAGTGTTACAAAGACCTCGAGGAGAAACTCTCGGCGGAATTGCCATAACTCTTCGGATTACCATTCTTGATGTGAATAATTTCCATCCTG TATTCCAACTCTCACAAGGGGAACATTACGAGGGCTTCGTGAAAGAGGGTACTGCAGAGAATACAATTGTGGAAGGGCTAGAACAGTGCCATGC AACAGATCGAGATACATCGGGGATACGAGGTTATTCCATCATTAGCGGGAACGAGAAAGGCTACTTCAAAGTCGAGACCGTACAGATCGGCAGC GGGGTAACGAGCCGTAAGTTCTTAGTGTTGAAGACTACGGGTAAACCGATTGTTCGTGATGATAATAATCCTTATATTATGCTGACGGTACAAGTCA CGGACGGAGGGAACCCCTCGCACTCGGGAACTGCGAACATTCGCGTGAATGTTGAAGACGCGAACGACCAGACGCCGGTGTTCGAGAGCTCA CAATACCGTGAAACAATAGCCGAGAATACTCCCATTCAGACCTCTGTGCTACGCGTACGGGCTACAGACAAAGACGACGGCACGAACGGCGGGA TCTACTATTACATGAAGAATCCTGTGAATAGCTATTTCACCATTGATGCTATTACAGGAGTTATTAGGGTCGCTAAAACGCTCGACTATAACGCCAGG GATAAACACACGCTGTACATTTCCGCACGGGACCGGGGGGACCCTCCTAGGACGAGTGCTGAAGCCACCGTAGAAATTTCACTGAGAGAGAATA TTCAGGGATGGCCGCTACCTGATAGCGCAGACCCGAAGGAAAATACGAAACCATATTTCCCCCAGTCCAGGTATACATTTAGTATTCGCGAGGATT TTCCGCCTAAAGGTGCATTACTGGTGATGCGCGCGGCGGACAATGACCCGATTGGACCAAATAAGAGATTACGGTACTCTTTGTCTGGAAATGGT GTCTCTAAATTTGCGATCGATCCTGAGAGTGGCGTGGTAACACTGACGGATTCTGTGGACTATGAAAACACCCCAAATAACCACGTGTACGATCT CACAGTCACCGCTACCGATCAGGGCCCAGGGTCTCTTAGCGCCACTACACAATTACTCATCGAAATCCAAGACGTCGACGAGAATAAAAACTCCC CAAGGTTTGACCCCCAGCAAGCAATCCTGGAAATTAGTGAAAATCTTAAACAGAACTCGCTAGTGACTACAGTAAGCGCTACGGACTCAGACAGT GTCGGGAACCCGTCGAGCCCTGATGGGAAAGTGGTCTACTCGATTGTAGGGGGAACTGGGCTTGGCGTCTTTCGCGTGGATTCAAATACAGGA GAGGTGAAAGTCGCTGTACCTTCTCTAGACAGAGAAGGTACATCCCAATACACTCTAGTTGTGAAAGCGAGCGATAACGCTACATTCCCACGATC GTCGCGCCTTTTTCTCATGATAAATCTATTGGACGAGGATGACAATTTCCCGTACTTCAGCCAGCCTATCTATATAGCCCAAGTCCCGGAGAACCA GCCGTCTGGGACCTTCGTCACAGTGGTGGTTGGCAGGGATGCGGACGAGGGATTCAACCCAAGCTACACGGTGATCACTCCAGGGGTTCCGT ACAAAATCGAGCCGTCAACGGGCGTAATCCGGACGTCCAAATCTTTGGACCAATCAGAGTTGAGCACAAGGGAGCTGCAGGTGATTGTTCGCGT TAGTACTGCCGGGTCCAAAACAGCGGACGGACAAGTAAATATAACAGTAATAAGCAAGGTCAACCGTCCACCCGTGTTCAAGAATACACCATATTC TGTGAAAGTCCCCGAGGAAATGGGGCCTCTGCCCAACTTATTCTGCATCGCTGCTGTTGACAGCCTTTCGAGGCCGGTCCAGTACACCCTAGCG CCGGGAGCAGATGGTCTATTCGAGGTCGATAAGGACTCCGGTCGACTGCATACCAAAAACTCCTTCAATTACGAGAACGTCGATCGCTACAATCT CCGGATCGAGGCTCGCACCAGCTCCGTCCAGGAAGTGGCGTCCGCCTCGCTCACCGTCGAGGTCACGGAAGAAAAAGACCTGCCAAAATTCT CTTCCGACTCGTACCAGCTAACTGTGGATGAGAGCGCTGCACCGGGGACTACCCTGGCTCCAGGGCTCCTCATCATCGACTCCGACACCTCCA GTGATCAATTCGACTGTTCTATGGAGGCTATCACCTCTCTCCACACCCTATACAACTTTGAAGTCACCCAACAATCAGGGCGTTGTTTTCTACGCG TTCAAGCAGGGGGAAAGCTAGACGCTCATTTAGCATCCAAGTACACGTTCAACGTTCGCGCGACTGACAGGAATTTCAGAAACATGTTTGCGAC AGCCCAGGTGGAGGTCAACGTGATTGACGTGAATGACCATAAGCCGGAGTTCTTGCAGGAGTCGTATTGGCTTTCGGTACCCAGTAGTACCCCC GCGGGGAGTAGTTTGGTGACGGTTCAGGCCGAGGATATGGATATTGGAACCAATGCGCAGGTTCGGTATGAGCTGCTGAGGCAGGAAAACTCA GAAAGGTTTATCTTGAATGACAACAACCAGCTGTCCACAGCGTCCACGCTGACTCCGAACGTCCGTTACCAGCTGCTTATACGCGCCTCCGACT CAGCTACAAGGAACCCCTCCTCAGCCCAGGTCCCCGTCTACGTCTCCGTGTACAGCCCAAGTGAGTCTCCGATCGTCTTCGACAAGTCTTCGTA CAATCAAAACCTTCCCGAAGATTCTTCTGCTAATACACTTGTATTCACGGCTAAGGCCACGAGATCCGGCAGCAGTAGCGGGATCACGTACGAAC TCGTCGGTGGTTACAAGCAGATCGGCGAGGCCATGTTTAGTATCAAGCCGGACACTGGCCAGGTCTACCTAATAAAAAAACTCGACTTCGAGAC CAAGTCATACTTTCCGATTGCTGTCCGAGCCAAGTATTCGGGAGGCGCCATCGAGCTCGCCTCCGAAGTAGTAGCCAAAGTCACAATCGTCGAC GTCAATGATAACGGCCCGAGGTTTGCATTCCATGAGAGCAGCAAGACTGTAGTGATTGACAGCTTTTCTGCCAAGGACACACAATTGGTCCAGG CACGAGCGCTTGACGCTGATTCTGGTAGCTTGGGCGAAGTGACGTACGGGATCGACGGTGGCAGAAGTACAAGTAACTTGCCTTTTAATATCAA CGTAAAGACGGGAATGATTTTCGCCACCAGGGAGATACTGTACACACAAGGGTCATCGTACATAATAATCGTCGTGGCAACAGATGGTGCCACGG ATGGTAGCCAGAAAATCCAGAAATACACCGTCAATGTCCAGGTACTGGACACTCCCCGTCCGCCCAGTTTCCCCCAAAAGACATATTCCGCACCC GTGACTGAGACTGCGGGAGTTGGGGACACCGTGACCACCGTCAGGGCAGTGTACAGCAAGCCTAACGCCTTCCTCAAGTACACCTTCGTGAGT GGGAATGAGGACAACACCTTCTGTGTCAACGGGTTTGGCATCATATCTGTTGCCAAGTCACTGGACCGAGAAAAGGTCGCTGGGTACACGCTG GGCATGCGGGTGACCTTGGGTCAGCACGTGGACGACACTACGGTGTACGTGAACCTCACTGATATCAATGACGACGCTCCACACTTCACATCCG CTATCTACAGGCGCTCCATCAAGGAAGGACTTGCTGAAGACACAGAGATACTGCCTCCTGTGATTGCTGTCGACCATGACTTTGGCAGCAACGG AAAAATTTTGTACTCAATCCTTTCCGGGGTCCATCCGGACTGGGACAAGTATTTCAATATTGATTCTGCGACTGGTAAAATCACGACTAAGATGACA CTGGACTATGAAACGCACAAGTCGCACACGCTGTTTATACGAGCCGAGGATAATGGCAGTCCCAAGAGGCTCAGCGGAATTGCTCAAGTCGACA

TAGACGTGATTGACAGGAATGACAACTCCCCCGTGTTCGCTGCCGCGTTCTACCGCGCAAAGATCTCCCTCGGTGCGGTCAAGGGGACGAGTG
TGCTACAAGTGCATGCTACTGATCTGGACAGCGGACAGAACGGTCAAATAACGTACTCAATCATCCAAGGCAACGAGGAGGAAGCCTTCACCAT
CAACGAACAGGGTGTCATACTCGTGGACAAGTCGCTAACAACGGTTGCTGCGGATAAGTTCTCGCTGAAGGTGGAGGCCCGCGACAAGAACGC
ATCTCCCCGCAGCGGTAGCGTTACTGTTGAGATCAACGTCTACCTTCCCGACGGTCCACCCATGTTCGTGGTTTCCCCGGTTACGGTGTACGTTA
AAGAGGGCGTCCCAGCCAATCATCGCGTCGCTGGTGTCAAGGCTGCCACGTCAGAGGCCCTGACGTACACTCTCTTGTCTGGAAACGAGGCC
GGCATGTTTAGGATCAACCCATCTACCGGTTCCCTGGATGCGACCCGCGAACTCGACTACGAGGAACGCAGGCGCTACGAACTGAAAATCGAAG
CACGTGACACTCGCGACCGCAGCGCAATGGTTGAGCTTATCATCATTGTCGTCAACATCAACGACAACAAGCCCACCTTTGTCGACATCGTCGA
CGGACAAATAGATCGGAAAGCCATCGGGACGTGCTCGCCGACCGACATTGCTGAAGGAGAAGTCATCACTCGGCTCGGCGCGTTCGACCGAG
ATGGCGACGAGATAACTTTTATAGTCCCGGATGATGTAAAAGACCTCTTCAAAATCGACTCCCGTGGTGTCGTTACTGCCAAGAAGCCTATCGAAG
ACTTACCGAAAATTTACCCGTTTAACGTCAAAGCTCTCGATAACGGCGAACCGCCACAGGAAACGGACGTCAAAGTAAGACTTGTTTTTGTTCATT
ACCGCCCGGACCAGAAGCACGTTCGGGTAAATGTCCGGGAAGACACGCCTACCGGGAGTGTTATCGCCACTGTCCGGAGGTACTTCCCTAACG
GCATCGTCTCATTGTTGCTGCCAGAAAAAGCGAATTTCAGCGTAAGGGCCAATGGTGACGTCATCTTGTTAACACCGCTGGACTACGAGAGCCA
GCAGTTTCACCGGATGACCGTCCGAGAGGAGCTTGGTAACCAGACAAATGATGTTGATGTGGAGGTTGTTGTTTTAGATGTGAACGACAACAGG
CCGATCTGGATGGAGCGAGAGAGACTGGCGCGAGTAAATACTAACAGCCGCGCCGGCGCACAGGTATACCAACTCGAGGCAAGAGACGAGGAT
GACGGTTCCAGTGGCCTTGTGGGATACCAGTTGAAATCTCCCTCGAATGATGCGAAACGCAAGCGCCGCTCACCTCCACAACATCCTTTCACCA
TTAACCCCAAGACGCGCCAAATGGAGGTGGCGGGCTCACTGAAAGATCAACGCTACGATTTGGACGTTTTCGCATTTGATTACGGGATTCCGCG
TTTAACCAGTGACACTATTACGCTCAACGTGGATGGCTCTGCGGCAGGGCAACTCCCGCCCAGGTTCTCAAAGGTCAGCTATCACTTCATGGTAT
CCGAAGACGCGAAGTATCTCTCGCTTGTTGGGATAATACTTGCTCGCAGCATCTCTGGAGCCCGATTGGACTACAAGATCGTCAGTGGTAATGTC
GGAGACAAGTTTATCGCCATGGGTGATGGACGAATCTTGCTGAATTCCTTGCTCGACTTTGAGCGCGACCAGACGCAGTATAATCTTAAAGTGAA
AGCGACCGAGCAAATACCTGATGGTTTGGACTCTACAGTAGACGTCAAAATCGATGTCATCAACGCCAATGATCACTTCCCGTACTTTGATCAGCA
ACTTTACTCGGTCCAAATACCGGAGAGTACTGCAGTAGGAGTCCTCGTGCAAGAGGTGACCGCTAAAGACTGCGACTGCCTCTCTAGCTGTACC
TGTTCTCCAGGTTTCCTTACCTATTCCATTGAACCCAGTAAAGAGCAAGGAGGAAAGTTCTACATTGATCCGGCAACGGGTAAGATTTCAGTCAGC
GTGGCGTTAGACTACGAGGATCAACGATACCACTTACTAAAAGTCTACGCAACGGATAAGGGAAAGAAGTCCTTCCAAGGACTGTGCTTTGTCAA
TGTTACCCTGACTAATGTAAATGACAACCGCCCTACCTTTCTAAAGAGCGCCTATGAGTTCCGCGTAGCCGAGGGCGCCGCAACCGGCGAATCC
CTCGCGATCGTGGTCGCAGTGGACGCAGATGGAGACGCGGTGACCTATTCAAAGGCAGGTGGTGCTTTACAATTCTCTGTGGATCAATCAACCG
GCGTGATCACACTGAACAGTGCTCTGGACCCGAACAAGAACCAGTATACCTTGCAGGTGATGGCCAAAGACTCTGGAGGACTCACAAGTACCGT
ATCCGTCACTTTCAACGTCGCAGATGCTAACAACAACGTACCACAATTCACCAACTGCGGGACAGTCAGCATCCGCGAGAATGACCCGCGGGAC
ACTAAAATCACGCAACTGACGGCTACGGACGCGGACAGAGGGCAGAACGGACAGATAACTTACTCCATTGAGGACGCCAGCTCACAGACTCTC
TTCTCCATCGAGCCCCGTACTGGTGTTGTCCGCAGCCTCACCAGCCTCGACAGAGAAAACAAGGACAGTTATAATGCAATTATCAAGGCAGAGG
ACGGGTCTTCGAAGCAAGATGAATCTGAGAGATTACTTTGGTATTGCTACTTGACCATTAATGTAGAAGACGTGAACGATAACAGGCCGTATTTCC
TCGCCGCTAAGTACTTCGGGAGCGTGTTTTCCAGTGCGCCTAACGGGTCCAATATTCTTACGGTCCAAGCTACTGATGCAGATAGTGGCTCTAAC
GCTAAGATCAAGTACGCATTATTAGACTCTGCTGGGGGGCTCTTCCGGTTGGATTCGTCGGGGATACTCAGAACCAATACTAATCCCGCCCGGCT
ACAATTGGAAACTGGGAAGAAGCTACTTTTGGAGGTGTCTGCTAAGGATGTTGAGTCTATCGCTGGGACGCAACCCGGTAAACCCACAAAGTAC
ACCACCCAGATCGAGATACTCGTGTCGAATGAGGAACCACCGAAATTTTCTCAACAGGTTTATACAGCGAGTATCAACGAAAATATGGAGACTGG
ATCTACGGTCACACGCATCACCGCCACTTCTAGCACGGGCGCAGAGATCAGCTACGAGAACGTTGACACGAATCCTCGCGCGAAAATTCTGTTC
CGCGTCCAGCCGGACGGGTATATCATCACGGGTGATCGGCCCGACTACGAGCGGGGTACCACCTACAACATGCAATTCGCCGCCAAGGACAAA
AAAACGTTGCTTTACTCCACCGTTAAGGTTGTCATCAACATCATAGACGTAAACGACGTCTCTCCAGCCTTCCTCCTCGCGATCAACACACGCAA
CGCGCGCGTCCTAGAGAACAAACCCGCGCCCACGAAGGTTATTAGCATGAAGGCCATCGATGACGATGGCAGTGAACCGCATCGACGCGTGAC
ATACGAGATGAAGGATAACCCCAATTTCCAGATCGACGCGAGCACGGGCATGATCACTACGAAGACTACTTTGGACCGCGAGGTGACGCCCAAG
TATGACGTCGAGGTGACGGCGAAGGACGGGGTCAACAAAGAGTCGGCCATTCTCTACATTACGGTTGTAGATCAGAATGATCAGCCTCCCGTGT
TTGCGCCCAAGAGCTACGCTATCAGTGTTCCTGAGGATTCACCCATAGGTACTTCTGTGTTAGATATCTACGCTACTGATGCAGACGTTGGCGAAA
ACGCAAAAATTACCTACTTCATCTCAAAGGGTGACCCCGAGGGCAAGTTCTCCATCGTCACTTCCCCCGTCAAGGGTGAGCTAGTGGTGAATGG
TAAACTGGACTTCGAGACAAAAAGCAGTTACACGCTTGAGGTGACAGCTACCGATGGCAAGTTTAGCGACACAGCTGTGGTTACTGTAACGATC
CAAGATGTGAACGACCTCCCACCCGTGTTTTCCAGTCCTCTGTACGAGTCTCGTATCCAGGAGAACACAGGGCCTGGTGCGGGCGTTGTCATG
GTAACAGCATCGGACATTGACAGCCCCACAATATCCTTCTCACTGGATGACCGCGGAAAAGACTACTTCCAGATCACGCCCATTCGTGCGAGTG
GCCCAGGCAACGTGTGGGTGGGTGACATTAGGACCGGAAGCAAGCAACTTGACCGAGAGGAGTCGCCTGTCAAGGTGTTCACCGTCATCGCC
AACGACGGCAAGCACACCGCACAGGCAGAAATCAGGGTTAACCTAACGGACGTCAACGATAATGCTCCAAGGTTCCCAGCGTCCCCGTACATTG
GATACGTAGAGGAGAACAAACCGTCCGGGACAAGCGTCATGTACATACAGGCTGTCGATGATGACGACCCATTGGCAGGCGGAAATGCCAAGCT
CTCCTACGAACTAACGGATTCCGCCGGAGACAAATTCAGCATCGACCCTCTGTCTGGACTTATCAAGACAAAGGTGACATTCGACCGGGAGCAG
ACTCCCAATAAGTTCAAGGTACGCGTCAAAGCGACCGACGCTGGAAACCCGAGACTATCTGCAAGCGTGGATGGTATTATCCATGTTTCTGACGC
CAATGACCACAAGCCGAAGTTTACGGAGAAATTTTACCGTGGTAGTGTTGCGGAAAATGCGCCACCGGGATACTCGGTACTCCGAGTAACAGCA
ACCGATGAGGATGTTGGCCCCAACGCGGAGTTTGAGTTTGTGGTGGTCCAGGGCAACGACCCACACGCATTTTACATTGACCCGTTTAACGGTA
CTGTACTGGTGTCGGGAATTCTTGATTATGAGAAAAAGAAAGAGTACACCATAACGCTAACGGTGGCAGACCGCGGAATGCCTCCCTTACAAGGC
GACGAGACCGCTTATGTTGTAATTGAAATTCTGGACGCAAACGACAACGCGCCGGAATTTATACCAAAGATATATAACGCATCGGTGCTGGAGGAT
GTCGGAGCTCGGCAGCCTGTGCTTACTGTGACTGCAGTTGACAGGGACAGCGGTCCTAACGGGAACTTCACATTCGCTATCGACCCCCGAAGT
GATCCCGACGATGCGTTCACAATAGAACCTAATCCAAATAATGCAAGTATCGGAATTATCCGTACGCGCGTACCTCTGGACCAAGAAAAGACTCCA
TCGTTCCACCTTAAGGTTACAGCTACGGACGCAGGTGGACTCCAGGGTGAGGGGGAGGTGAGGATTAATGTAATTGACGTCAATGACAATGGCC
CTTGGTTTGTTCCCCCTTTCTTCGTTGGCCAAATCAAGGAAGGGGTGAGTGCGCGTCAGTTTGTCACCAAGCTAAAGGCCTACGATCCCGACGC
ATTTGTCAAGGACCAAGTCATCACCTTTTCTATTTACAACGGAACAGTGGGCGAAAACTTTAAACTAGACCCCGTCTCTGTGACTAATGAAAGCGT
AGACTTGCATTCTTACGGTGTCTTCGACCGCGAGGCTGCTCCAGTGTGGAAGATCGGCATCGAGGCTGTGGATAACGTTGGGCCGAAACCTCA
GAAGAACTTTACATACGTCTACGTTGACGTTCTAGACGTTAACGACAACGCACCAAAGGACGGCAGCTTGCTTATCATTGTCAACGCCTATGACG
GGAATTTCACCGGAGGTGTCATCGGGAAACCATACTACCAGGATGATGATTTTGACGGCGACGAAAACACCTATGAGCTCAACTCTCAATCGCCG
GGTAGCTACTTTAGAGTAAACGAAGGCAACGGTGACATTACGGCCGCCCCAATGATACCCATGGGGGAGTACAACCTCAAGATTCGTGTTACCGA
AAAGAAAGACAGCCCAAGTACTGTAACGTCATCTGTCCGAGTCCTAGTGCGGCGAATAGACAAAGAAGCTGTCGACAATGGAGTAGCAGTTGAG
TTTACGGACATGAGAAAGGTTGGGTACTTCGTAGGGGATTACTATAAGGGCTTTGAGGACGTGCTTGCTAGCACTCTGGGTGTACCTACTGGAGA
TATCAAGATATTTAGTGTGCAGAAGGCTCACGATAATGGACTTGCTGTAGTTGTGTTCTTTACTGTTGCGGCGAAAGATAGTTACATGCCTCATTGG
GACGTTGTATCCAAACTTGTTGACGCCAAGAAACCGCTAGAAAGCCTCGGGTTAAAGGTCAGTCGACTAGGAATGGATGAATGCTCCAAAGGCA
ATGTCGGTCAAAGTGTTGGCGTAGCCAAGAACATCCTTGTACGGTCATCCAATTTCTCCGTGGCCAGCGGTGATTACGGAAAAGTTCCCGCCCC
AGCATCCTCTCTGACTATCGTTTCCATAGACATCTTACCCAAATGTCTCTACGAGGCCGTATTCCCACCTGAAAAGCGCTGTAAGCCGCATAACCC
GTGTCTCCATGGTGGCAAGTGTTACGAGACGGTGCCTGATTGCCCAGGCTTTGTCTGTAAATGTCCCACAGGGTACCATGGTCCCTTATGTGAG
ATGACAACACGCACATTCTACGGCAACTCGTACATTTGGCTTCCAAAACTCATGACATACTCCTTAAGTGACCTCGAGTTCGAGTTCATGACGAAA
ACGGCGGACGGTTTGTTAGTCTATCAGGGACCTGAGCGTGAAGGTGCAAATAACGGCCTGAAGGACTTTATTGCCGTGGTGCTGCGAGGAGGT
CGCGTAGAGCTCTTTGTTTCACTGGGACTGGATCCTGTGACCGTTAAGATGGATAAGGGACCTAGGCTTGATGATGGAGAGTGGCATACTGTGC
AGGTCCTCAGAAATATGAAGGACATCGAAATCATAATCGACCGCTGCTCAACTGCTTTGCTTGAACACAAACCAGATGGCACGGTGGTGGAGAAC
CGCAAATCGTGTCACGTTTACGGGCGCATGCTGGGAAGATCTGTCTTTCTCGATGGCTTCGGTCCACTGCAGATTGGAGGCGTCTCAAACCCAA
ATATGGATTTCCCCGACATACCATATACGGGATTCAAAGGTTGTGTTCGTAATATCAAAGATAATCATAACCTGTACGATCTTAAGAACCCACTTAAA
GTCGTGAATGCCCCAGAAGGTTGCCAGCTCGCTTCTGCTTGTCCTGAATGTAAGAACGATGGTTACTGCGAGCCGCTCATGGCTAGGGACAGCA
TTTGCGTGTGCAACCCTGGATACAGCGGGAAACACTGCGACGGACGCGGTAAAGCGAGTTACTACCTGGCCAGTAGCTTCACTGAATATCTGGT
TGCTGCCCGTCGTCGTCGTCGTGAGGTTGTTCCACCACCCACTGAGATCTTCAATAGGTTCTACACCACCTTGGCGTTACAGGTTAAGCTGGAT

GAGGACGCTACGAATGTTGTGGTATTCCTTGCCTCCAATCGCATGGGAACAGAGTTCCAGAGAGTGGACGTTAAGGACAGTAAAATCCGTTACGT<br>GCTTCGCTTGGGCGCACGCATGCTTGTATTAAGCTTTCCGCAGCTCAACGTGACCGACGGCGTCTACCACAGCGTGATAGTTCGCAGGCATGGA<br>GACTATGCGATCATGCAACTTGACTACAGCGGCTACGTCATCGGCAGCTTGCACAGTCAGCGAACCCTCCTGGACATGAGTGGTGGAGAGATAT<br>TTTCCGGGGGTCTCCCTAATATCACAATCGTCCGTATCATCGAAGCGATCGTTGAGAACGACGGGAGTGCTGTGATCAGCACGAACGTGCGTAA<br>CGATGGAGACGGTTACGCCGCCGACGTTGGAGGGGTGCATGTCCGAAATTTGCAATTGTCTGGGCCCCTTAACCTGGTGTCTCGTAAAAGGAG<br>AGCGTCTGGCACAGTCTCCGTGCTAGGAGACTTTGGAGGGTGTATAGCAGGGACCAGTGTGAATGGCGCCAACATGGAGAGTGACCCGTCCAT<br>CAAGGTCCATCGGCAGAACGTTCTAGACGGATGCCCATGTCTCTCTAATTTTTGCGCAAACGGAGGGACGTGCGTGGACGCTATGCCGCCCTAC<br>TGTATCTGTGCCCCAGGCTGGACCGGTCCACTGTGTACCATAGTGGTGACTGCCCCACCCGTCGGTGAGCGAGGCACGCCGTTCATGCACCCT<br>GCCGTCATCGCCATTATCTTAGTCGTCATGCTCGCCATCTTCATCATCATGGGCGCCGTCATCCTCAAGCGACGTCCTGAGCCCGTGGTCGTCTA<br>CGCTGACTCCACCGATACCGGCCACGTGCATGACAACGTGCGACTTTACCATGACGACGGCGGCGGGGAAGAGGACAATCTCGGCTACGACAT<br>CACCAAGCTGATGAAGTATACGTACATAGAGACCACTATAGCGCCGCCAAGTGTAGCGCCTTCTAAGGCATCCGAAGACAAGATATCTACCAGCT<br>CGGACCAGCCCCTGCTCCAAGGAAGGCCCCCGGATGCCGTGTTTGGCTTAACGGGCAAGGAACCAGGGCCCAAGATGCCCAAATACATGGAG<br>GGAGATGACGTGGGAGACTTTATCACAACACGAGTCAAAATAACCGATAGAGAGGTGTTCTTAGCTGTCGATGAACTGCACATCTACCGCTACGA<br>GGGAGACGACACAGACGTCGATGATCTGAGCGAGATCGAGCCAGACGAAGAGGATGAGGAATATGAACAAGAGTTCGATTTCCTCAAGCAGTG<br>GGGACCCAAGTTTGACAAACTTGCAAAGCTGTATGAAGACGTGGATGAGTAGTAATAGGGGATTTTTAAAATTGACATTTATGGAGGTAAAAAAGC<br>GGTTTTGAGAAGCACATCATTTCATGTCAGCAACATGCCACTCGATATCCTGATGGACTCGACGTGTGCAACGTTATAACGGCGACATGGAAGGT<br>GTCGTAGGCGTCACGTGGGGGATGCATGCAACTCAGCGAGCTATGTGACAATACGCGCATAGTGAACTGCTGGTTGCAGCGCAGTGTTGCTGA<br>ATTCTTCCTATTTACCAGAAGGCCATCCTAAAGAGTAATCCCATATAAAGTTAAAGTTCTATCATAACTAAGACCCTATGTATACATCCAAAAAATTCAT<br>TGTACATACAGACTTTCTTATCTAGAAACACCTATTTTTCCATGTATTAGTTCCTTTTTTATAGACGCTTTTAGGAATGATTTTGATGGCATTGTTTGAG<br>ATGTATAATGCTATGTGTTTTTTGCCTCTCGAACAGATTTTAAACAAACAAAAATATGCATGGCGTGTGCTTTTAAAACATTTACTGAATTTGCGTATT<br>AGATAGGGAATGTTAATAATTCGACTACTGTTGGTAAATGCGTGTGATGTCGGTTGAACTTTTAGCCACATTGACTTTGAAGCAAACGGCTATTAGT<br>CATGTGACAGTAATGATGTCACTGCACTGGTTTTTCATTTGCCCCCCCCCCCCCCCCCCCCCCGATTGATTGACAGTTCATGCTTGTGCTTTTCA<br>GTCAAAGCAGTTTTCACCTTCAGACCATTTCACCCTATTCTCAATTAAATAAACTAAACCAACAAGTTAACGTAATTATGAATATTCTTGTGAATATTC<br>ATGAGTATTTCTTTCTACAGATTGATGCCCATAGTTTGGATTTCTCACTTTTAGATGGTTGCGGTGTGGCAATGCATCTTGGTCAGGGTGACTGGG<br>TAATCGATTAACTGTCCCTGTACTATTTAATTAGAGATGTCCATAATTTTGTTTAAAATAGAGAATATTTTCCAAGTAGCTTAGACTTGGTAAAGGATTG<br>GATGGACACGTATAATATTTTGTATTATTGTAGTTTTGTATTGTACAATGGTAAGATTTG
**Brachyury (5‘ to 3‘)**
GTAATAGTTTATTAGTAACCCGAGCATTAAGAGCTCTTAGCCAAGCCGCCAGCAAAAGAACAAGCATAGTCGGACATGCACTCGGACGAGAAGAA<br>ACCTTTCACCGTCAACGACATCCTACAAGCTGCCGAGAGTACCATGGCCAGTACTGATGACAAGGATCTGGGAGAACATGCCAAGAACACAGAG<br>GACAGCCAAGTCAAGATCGTCCTTGAAGAAGCTGACCTCTGGAGGAGATTCAAATCCTTGACCAACGAGATGATCGTCACAAAGAATGGACGGC<br>GCATGTTCCCCGTGCTGAAAGTAAACGTGACAGGCCTCGAACCAAAGGCCATGTATTCCTTTCTCCTGGATTTCGTCTGCGTGGAAGGACACAG<br>ATGGAAGTACGTGAATGGGGAGTGGGTTTCAGGCGGGAAGCCCGAGCCGCCTACTCCGAGCTGCGTCTACATTCACCCCGACTCGCCTAACTT<br>CGGAGCGCACTGGATGAAACAGCCTGTTGGCTTTTCAAAAGTCAAACTCACAAATAAACAAAACTCTGGGGGACAGATCATGTTAAACTCTCTAC<br>ACAAGTACGAACCCCGGTTACATATCATAAAAGTTGGCGCGTCGGATAACAACCGTACAGTCGTCTCGCATTCTTTTCCCGAGACTCAGTTTATAG<br>CAGTCACCGCTTATCAAAACGAAGAGATCACGAGTCTAAAAATAAAGTACAATCCGTTCGCTAAGGCTTTCCTGGACGCTAAAGAACGGCAGGAA<br>CAAAAGGAGGCTCTGGAGCAAGCGTCCGAATCGCACTCAGCTTACTCCCAATATGGATGGTTCTGTGCAGGGTCCACGCCGGTCTACCCGCAT<br>CATCATCACCCTTCCTCGTATCCTCACATTCCTTCATCTCCGTACAACAGAATAGGCCAACTAGGCATCCGAAGTCATCGACCTGCGCCCTACCCC<br>AACCCATACCATAAGCGGACGGATTTATCTTCTCCTGGTCACCCCCAACAGTACTACGCCACGGAGTCGAATCAACTTCTCTTTCCCGCCCCAGC<br>TTCCATTGAGAACCTAGCTGTGCCAAGCACCGTCGCCCATCACTCACAAAGCGCGGGAATCCACGCCGGGCTCACACTCTCACTTAGTAACGCT<br>GCTTACGGACGCCCAGGGCACGATGAAGCCATGAATCGAAGAGAAACAGACAAATCCAAGTTTCTGCAAAGCGGGGGGCTGACTCTCCATTCA<br>AGCTGGAACACCATACCGCAAGCTTAAAGTACAAGGACTCAGAACAGTTGTTCTTGTATATATTGATTGAGGGCGTCTGCCAAAGTCATAATTTAGT<br>ATATTTGCTTTTTGCCCTGCTTTCAGAGACCTTTTCGGTATCTCGCACTCTGCAAGCAGAGGGGCCTTTATTCAGCGACAAGGAAATGGGGGTAA<br>CAACTCTAGAATTTTCTGATCAACTTCTAACTTTCAACCGGAAGTGGCTATCTGTGCGGTTTTTTGGGGGGACGGATTGTTAGTGTACAAGAAATT<br>TAAGGAAAGAAAATAAATTCGTCCAAATGACGCTTAACAAATTGTTAATAGATATCGAAAATACGAATATTTAAATGATTTTACAAAAGAAATAACTCG<br>ATGATTTAGAAAAAAATTGTAAATATTATCTGTTCAAATCATTTTTGCATTGTTCTTTGATTCTAAATTAGACTGTATAGAAAAGCCTACTTCAGAAAGT<br>AGGGAATTGTATATTAATAAAAAGGCGATCTTATATGGCGCCATCAATATTCAGACATTGAATCGCGTTAAATAAATTGTTG
**Six3-6 (5‘ to 3‘)**
ACAGTGGGTCCAATTGGTTCGTCAAAGCCTTCTTCGCCCGAGTGTAGAGTCTCCTTTGTGCTCTGTCATTACCATATGACAGTCAATGCAATTAGT<br>GCATTACAAGTACTAGTTAAGCGTAATAGGCTGACTTGATATTGTGTTTTATATTTAGTCTAATTCCTTAGTCTGTTTGTTTAGTTATTGTTCGAAAGC<br>AAGCCCCCCGAGTTGAGATCTTTTATTTCTCGGGAGTAGAGCACGCCCCGACTCGCTACTATTGTTGGAGACTAAAAGCCTTTACTTAATGTAGTC<br>AGTTCTCACGTCTGTGACACTGTTTAGGTTTGGAGCAATCCCGTGCAGCTTATCCCGAGATCAAACACCATCCGCTTTAATAGAGTGCATCAGCC<br>GGCGGACGATCACATCTGACAAGTCTTGCAGTGCGGCAGTACTGCGACCGCGACCATGTTCGCACCGTTACCAGCGCTCAGCTTTAGCGCACA<br>CCAAATCGCCCAGGTCTGCGAGACGTTAGAGGAAAGCGGAGATGTTGAACGCTTGGCCAGATTTCTTTGGTCCTTGCCTGTGGCTCCGGGAAC<br>ATTGGAGGCTCTGGGGAAACACGAAAGTGTACTCCGGGCTCGCGCTATAGTCGCATTCCACATGGGAAATTTTCGAGACTTGTATCACATACTAG<br>AGACGCATCGGTTTACGAGGGAGTCGCACGCTAAACTGCAAGCGATGTGGCTTGAAGCGCACTATCAAGAGGCAGAACGTCTGAGAGGTAGAC<br>CGTTAGGTCCGGTGGACAAATATCGTGTTAGAAAAAAGTTTCCTTTGCCCAGAACAATCTGGGACGGAGAGCAAAAGACTCATTGCTTTAAGGAA<br>AGGACTCGGAGCTTACTGCGTGAATGGTATCTACAAGACCCGTATCCGAATCCGACTAAGAAGCGCGAACTAGCGCAAGCAACAGGACTGACTC<br>CAACACAGGTCGGCAACTGGTTCAAGAATAGGCGGCAAAGAGATCGAGCCGCTGCTGCTAAAAACAGAATGAGTCAACAACAAGGGACGGATT<br>TGAGTCACTGTAAACCGCCTTTGAGTCCTGACGAACTTTCATTGGACGATGAATGTAGCGACCTCCGCGAGCTAAAAAATACCCCGGTGTCATCA<br>CCAAACATCACTAAACTCTCCGAGCAGCGGCCCTGACTTGCTCCGCCTTGTTAGAACTAAGAGACGATAACAGGGCGATAGAAGGAGAATGACT<br>TAAAATAAAATAAAATAAAAACTCCATTAGAATAATACGAGGAAAAGTTGTGTACATGAGTAGAAGATTTCGAAAAAGGAAGAATATCAAGAAATATAT<br>GTAGCTTTTAGTGCCTTCGGAAATCAAGTCACCTGTGGCGACCACGGATCGGGGGAGAACAGACGGGGACACATATGTAGAATTAGATTGCGCT<br>AGACACGCACTTTTACCGGCTGTACAGAAAAAATAACATATTCTTGTAAATTGTTAGAAGATCTTAGGTGTTAGAATGCCTTTTAAGTCAGGAAGATA<br>AAATTCCCTGTTAATAAAACGTAGCTTGTATTTTGGGCTGTTTTAAGCTCTTCGGTTGTACATTTTTGGGGGCTAAGAGAAAGAACAAATAAAAGAA<br>AAATAATCAGCACACCTCTG

Table S2. Submitted sequences for HCR probe generation

Group	singleR.labels	newIdents	avg_score	rank
Mesoderm_agg.4h	Mesoderm	agg.4h	0,50482666	1
Mesoderm_agg.12h	Mesoderm	agg.12h	0,49179268	1
Mesoderm_agg.48h	Mesoderm	agg.48h	0,43979358	1
Mesoderm_agg.24h	Mesoderm	agg.24h	0,38139352	1
Mesoderm_wt.24h	Mesoderm	wt.24h	0,34019005	1
Endoderm_agg.12h	Endoderm	agg.12h	0,10119391	2
Endoderm_agg.24h	Endoderm	agg.24h	0,07551296	2
Endoderm_agg.4h	Endoderm	agg.4h	0,0665422	2
Endoderm_agg.48h	Endoderm	agg.48h	0,04158794	2
Endoderm_wt.24h	Endoderm	wt.24h	-0,0092309	2
Ectoderm_agg.12h	Ectoderm	agg.12h	0,00679617	3
Ectoderm_agg.4h	Ectoderm	agg.4h	0,00313975	3
Ectoderm_agg.24h	Ectoderm	agg.24h	-0,034524	3
Ectoderm_agg.48h	Ectoderm	agg.48h	-0,0389231	3
Ectoderm_wt.24h	Ectoderm	wt.24h	-0,0749227	3
Neuronal_agg.4h	Neuronal	agg.4h	-0,0346418	4
Gland Cells_agg.12h	Gland Cells	agg.12h	-0,0404037	4
Neuronal_agg.24h	Neuronal	agg.24h	-0,0501314	4
Neuronal_agg.48h	Neuronal	agg.48h	-0,0557535	4
Secretory Progenitor_wt.	Secretory Progenitor	wt.24h	-0,0923819	4
Secretory Progenitor_agg	Secretory Progenitor	agg.4h	-0,0385637	5
Neuronal_agg.12h	Neuronal	agg.12h	-0,0515581	5
Gland Cells_agg.48h	Gland Cells	agg.48h	-0,0564588	5
Gland Cells_agg.24h	Gland Cells	agg.24h	-0,0645815	5
Cnidocytes_wt.24h	Cnidocytes	wt.24h	-0,0982116	5
Gland Cells_agg.4h	Gland Cells	agg.4h	-0,0498119	6
Secretory Progenitor_agg	Secretory Progenitor	agg.12h	-0,0543732	6
Secretory Progenitor_agg	Secretory Progenitor	agg.48h	-0,0755878	6
Secretory Progenitor_agg	Secretory Progenitor	agg.24h	-0,0759984	6
Neuronal_wt.24h	Neuronal	wt.24h	-0,0993192	6
Cnidocytes_agg.12h	Cnidocytes	agg.12h	-0,0758632	7
Cnidocytes_agg.24h	Cnidocytes	agg.24h	-0,0844906	7
Cnidocytes_agg.4h	Cnidocytes	agg.4h	-0,0863492	7
Cnidocytes_agg.48h	Cnidocytes	agg.48h	-0,0913767	7
Gland Cells_wt.24h	Gland Cells	wt.24h	-0,103571	7

Table S3. Cluster ranks based on projected module scores

Beta-Laminin (NV2.1719) promoter

GAGGCGACGATGACTGTTCTATTGTGTTGGTCCTTTGATCTTAACTCTCCATGCTGGACGATCCCGAGCAGTGGTTTGCCAAG
AGTTTGAGCTTATATCCCTCCACTTCAGGTTCCTCTTTACCACATCTTTGAACCTGAGTCTAGGCCGACCCTGATTCCTGGTCC
CTGTCGTGAGCTGAGAGTAGAGCAGCTGCCTAGGCAATCTGTCAGTGTCCATGCGGTGAACATGCCCAGTCCATCTGAGATT
TTTCTCAATGAGTGTGTCAGCCATTGACCGTAATCCGGTCCTCTTGTATATCTCTTTGTTTGTGACTTTATCCATCCAAGTAATTT
TCATTATTTCTCTCAGGTGTCGCATCATATATGCATGTAAATTTTTCACCTGGCTGCGATATACAGTCCATGTTTCTGCACCGTAT
ACTAGGGCAGATAAGACAACTGCTCGATAGACCTTGCCTTTAACCTTGAGCGATACGTGATGGTTGTCCCACAGCCGCTCTCT
CAGCTTAGCATATGCTGCGCTGGCCTTCCCCTTTCTGAAGGTGATCTCATTATCTATTCGTGCATTGTCAGAGATGGTACTTCC
CAAGTACACAAAGTCTGGAAACAGTAGGGCGCTGAATACTCATTTTATCCTGAGAAACAGTCCATTCGACGTGAAAAGCTGAA
GTGATAAGAAATTTTGTTTCTTAATTTTCGAATAGTGTGAATTAAATGAAGTGAGGCCCGTATTTTTCAACTGGCTTGCTTGAATT
TTTAATTTCCATGCATACTTATTCTGACTAGTCTGTCAACATGGTTCTAAAACTTGAAAAAAATGATTTCCTTTTTTACAAGCAAAA
TCTGACCGACTTTCCCAAAAAAAGCCTCAAACGTGACGTTATATCGATCGGAGCTTATTAACCCATTGCCTCTTAGGCTGTACT
GCACCCTGTACCCTGTACTGCTCGTGGATAAATTAAACAAGAGCCTCCAAAAATCATAAATGCAAATATAACAACTAGATCCAGA
TACTCAGGCATTTAGCCTATGGGATATTAGGCTTTTTTTTAAATATACAACCCAACTGTGAGATGAACTACTACTGTACAGCCAAA
TATTGTAGCTCAAGTTCTTCGACAAATTCCCAACTAGACAAGAAAAACATGTTCGAGTCACAAGTTTTTATCTTTATTTTGCCCA
CCAGGTTCAGCAATCTCATTGAAACTTATTTGTAAAATTTAAACTTTAAAAACTTAATAAAGTTTTTATCTTTATTTTGCCCACCAG
GTTCAGCAATCTCATTGAAACTTATTTGTAAAATTTAAACTTTAAAAACTTAGTATTCTTTAAAGCTCATCTGTGGATCGCGCTAAG
TATACGCGCGTCTCGTCGCCGTCGAAATGAGGCCAGATAGAAAAATCCACAAGTCAAAAAAGGTCAAGTCAAAAAGCCGCGC
AATGTTTTCCAGAGCTTGCATTCTTCAATTTCAGATTGCGCTTTGTGCATTGTTTTTTCGATAATGCTGTGCTTAATCAGACACC
CACAAAGTAGTCAAAGTGTTTTTGTTTTAAACTGCAACAGTATTTAATTATTATTGGGTGTGTATTCGTAGATGAGACATTGATGT
TGAGAGCTATCTGTATAACCGCTTTTTTTCCCGTTGCAGCTCATTGTTTGTATTGTTTGCAAGGTGATAGGGCGTGTTTAGGTAA
ACCGGGGTCAACATCCGGGAGTCCGTTAAACACGGAAGCCCTCTTCAGCAACCCGCCAATCAACTGATCTTATTTATACTAGC
CTGACTTATCTGGCTAATAAGTCTTAACCAATGTATTGATAACGTCTTAAATCCAGCACTGAAAGCGCCAAACATATTTATAGAGC
TTGAAAGAAACATATAAGAAATACTCGTGACCGCGAGAGGGTCTCGATTCACGCAAACATGAATGCCGTCCCGTTCGACCAGT
GGAGAAACACATCCTGACAGTTCACGGGAAACGATGACACAAATCTTGCGTGACATCACTCCATTTGAATTAGATAGATATATC
GTAGACGATCTGTAGAGTTTACAAGTCGATACTTTCCGCACAGTTTAAGTCAAGAGCAAAGTAAATTCTAAGAGTTATTACCTTT
AGTTGTATCGTCAAACCAATTTGTCGCCAGTAAGAGGATAATGTTGTGTGGTAAGTGATCCGCTATTTATTGATCAAGTTTCTTA
AAAACTCGCTAAACTTGCCTCAGGGTGAAGTCAGTGAGATTACCTACCAACAGAACTCAGATGATTACGGAAAGTCCATACCT
CTTGGGTGTGTCAAAATAAAATGCGCCACTAAGAGATTCTTACTCTGTGCTTTGTGGGGAAAGACACGCAAGATACCCTCCTC
GCTTCTTATTTGATTAGTATGTAACTCTAGACCCGAGTTGTAAATGTAAGCTAACACCCTACCCTCCCAAGGACCAAGATCTTCG
TGGGGAAATCCTCTAGTAGATTTGACGCCATCCGATTGGCTGGTAGTCGCATTGCTAACATTCATTGACGCGTATCGTGTTTCC
GGTTCACTCTTGCTGCCTTCCACTCCTTGTAACTTGGGCTTTACGCCAGTCCGTTTGGTCCTAAGTGTGAGGATTCTGCAACA
ATACCGGTGCAGTGGAGTCGAGGGAGGTAGCTAGGGCGTCCTGGAAGTGCTACTGGACTTGCTGGCGTAGAATTAGCTTGTT
GGACTGGTTGTTTTGTGAGGGAACCCCGTCATATAGCAAGCCTTGTCTGGTCGATCGCTTAGGAAGTCTGACTTCAATATTGC
AGCAAATCCTACATTATATTTTACCACTGTGTCCGTGAGTGAATTTTGAAAGGTTTGTTTTTCTGCCTACAACGACTCGACTGCT
TATTTTGGGCTAAGGTTTGTCTATCTCGAAAACAAAGTGGTCTTTGTGGTGGTAAATGCTTATATACTGTGCCGGCTATGGGTAA
CAGACCCTAAGCTGAAATATCAATTCATCAGATAACTCTCTTCAATGAATTGCTTTCTCAGTTCGGTTTTGCATCCGTCACACAA
GTCTTGTAACTTATAGTTTGCCTGATATTATTACAGATAAACAGATAAAGCTCAACAGTGGAAATACCCAAAGGGAAAATTTGAGT
TGTTTACGAATTAGAAACCCACACAAGACGATTCAGCCCGGCCATCCTCAGGTGTTTCACAACCTTCACCATGTTATTCGGTAA
GTAAACCTGCTACGCTTTCTATGTTATTTTGTTGGTTATATGACTCCTTAATTTTTTCAACCTGTCCTCTGAGTGGAAATATTTTTT
TCGTGCTTTGTTTACAAAATTCCAGGTATCACCGTTTATCGATCTTGAAACGCGAGTAAAAAATTCCTTGGATATTTTCTATTCCG
TGGTTTTCCATCCCTAAATCAAACGTTCATCAATGACTCGAGAATACATAATTCTAACCAGACAGTAGACAACTTAAGATAGATTA
CTAAACCCGATAATTATATAGTTGAACTTTTATCTGCGCTTGCTCTCAAATGACGTTTCGTCTTCTTGCTATTACTGTCACTGTTTT
CCTCCCGTGACCGCTCTAGTACTTCAACAAACACCGATATCTTATATCTTACGTTTGTAAAGCATGGGAAATCACGATTCTATGG
ATAAGGCCATAAGGAATGCCATATAATTCCTATAATAGCGCTGAAGTATGGTGAATATCACGTTATGCAATCTTGACCACATAAGG
CGGCATATCATGTCTCCATCTCGTTATTTTACACAAGTGAGTCTCGCTATATTTTAGAGACCTCCCGGCCTGATTCTGGCTCAAG
TATATGTTTGTTTGTTTTTACGAAAAATGAAAAAAATGTTAGGGGAGCCGGAACTAAGTTGAAACTATTCACTAACCAATCAAATG
GCACTATGTTTCGGGTTCCGGAATAAGCGGCTCCGGAATATAAAGTTGTAAAACAACTTGTGACTGATATTGAATTTCCCCATAA
CTTCTCATTAATGATTTGAGCAAGCTGACGCTCTATTCTCTGAATTGTGTTACTCAAGCATAAAATATACTCAACAGTAACCCAGT
ACGCGCTGACGAATATGTCCAGGAGGATAAACAATTCGATATAATTTTTTTACATGAGTACTTTAGATTTAAACCGTCAAGCCTTG
ACAAGGTCTGATGTCATGCACTTAAACCCCAGAGAACCTGCAAAGATGTTACCCCAGATGATTGTTTTAGTACTCATCTAAGTG
CACAGGTCGATAACTGTGTATGAAGCCCTAGGGAGGGAAAACACAGAAGTGCGCGCGTACATATGGGACCGCAAAGAAATCA
TGGAGGGTTGAGGGATTAGGTGACAGCCAAAAACCCTTCCCAAAAATCTTCATGTTTATCAGGCCCTAGGGAGGGAAAACAC
AGAAGTGCGCGCGTACATATGGGACCGCAAAGAAATCATGGAGGGTTGAGGGATTAGGTGACAGCCAAAAACCCTTCCCAAA
AAATCTTCATGTTTATCAGGCCCTAGGGAGGGAAAACACAGAAGTGCGCGCGTACATATGGGACCGCAAAGAAATCATGGAG
GGTTGAGGGATTAGGTGACAGCCCTACCCCGCAATGTCCAATGCTTCATGTTTCCGATTTTCAAGCTAGCTCACTAACACTTCC
AATGATCCATGTCTCCTTAG

FoxA (NV2.11441) promoter

TAGACGGTTTGGCGCGTATCATACGTGGCTAAAGGACCTGTTTGGCATTTTGTCCTTGATAAGGTTCCCATAGACTGGGCAAA
CACACTGTTTGGCAAGAGAAAATATCAAGTTACTCACATTTGCCCCACACAAAAAAGTCACAAATTCAATCGGCTACCTAGTCC
CAGGGATTTCTGAAAAACCAAGAAATTACCATTACTATATCCATCACATTGTCTTTATTAATTCGAGGGCATATACGCCAAAATTAT
CTCGAAAAGGCTAAATTTGTTAAATGTTGTGCAAAATCAACAGTTTCTGGATATTTAGTTTTAGAAAAGCATAAAATGGATATTCA
CACGGCCGAGACTAGCGTGCCCCTTTTGCCACTTTACTCCATTCCCCTAAGCCTGAAGTAAAGCTTTCTTAAGCCCTGTTAGTA
CCAAATCTAATTTGTTGTGGGTCGTAGAGAGGACATGTTAAGCCGGTGATGGTATTTTCGAAAGGGTCGTGAGTTGTGTATCAT
CGTCAAATTATCATAAAGTCAATTAAAGCTCGCTGAGAGGTCGGCGCGTTTGAAGAGTTTACTGCGATTGCGCTTTTAATAAAAT

AAAACCCTTGCTCAAGTTCTGTAAAAATTAAACAAAATGAATGGTGATCCTTATATTGGGCATTGGGGAGACTCGGTTTCTTATC
TTCCTCTTTGCATTTTCACCGAAGGCTTGTCCTTTCTTAGCTATTCGAAATTGATTCGAGACTACACCAGCTTTCCCGAGATAAG
AAATAAACAGCTCAACTTAAGGAACTGCTGGATTTTTCTACTCAATATAAAGTTTATTTTGTGAGAAGAAAGTTTGCCACAACATC
GGGCGAACTAACTTCATTCTTTGTTGTGGCTAAATTTCTATCCTATGCAAACATTTATCAAAATGAAAACCTATCAGATGGCATAT
GCCACTCAAAAGTGGCCTAACTTGTTTCACTTGCGCAAACAAACTTGTGATCTCTGTGCTTCTGGCAGAAAATAATTTAGATTA
ACTTCTAAAAATACACCAAGCTCTTAATAAGAACTTGGAGTGAAAATAACGTTTGGTTTTTTGTTTTAGCGCACTGTGGGAAATC
TGGGCGTTCCCTGGTGTACCAGAAAGAAATCGAGGATGAGTGGGTAGTTATAAAACATTGAACGGCATAGTATTGAGGCATGT
GTGTATCCTAAACTACCAGATAAATTGGCGAATGTAACTAATAGAAATGTTTGGTTAACAAAACCTTGTGTTGATCGTGTAAAAAT
TTCAATATTAACTCTCTTTATCAATTATAATAGTGGTGCTTAGCTTTGGACAATGCCTCAGGGTGGATTGTGAGTAACACGAGTAC
ACGAAGCACGGATATCTACGGGTACAGCTCTCGTTGATACCAGCGACCAAATAAAAGGCGCCAAAAGACGTTTTTCATCGTTG
GCGAACTTTTTAAGCTAATGACTGGACTGTATGCGTCTTGGGAACACAAAGTTTTAATTTCGGGCTTCCTTTTATTGTCACTCTC
TCTTGTTAAGGCTGCTTCGTGGCAAAACCGCGTAACGTCTATTTCGATCAGCGTTTCCATTTAGCGCACGCATTTGCAAAGCG
CATGCGACTTTTCTCTGAGGATAGTCCTCTGCTGCTAAGTGAAAAGCAAAGGTCCGTTTACAAACATTCAACAAAACACAAAAA
GAAGAGAAGAGATAATACTGATAATATGAATGTAAAGTTTATACGGCCAAACATTTACAAAGAAACAGCGAGCCCTTTTGCCTCT
GAATGTATCATCATGGTTAACCAATCACAAGCGCGAACATTAGGATAAGTAAATATTGCGTTCGCTCTGATTGGCGGAGCGCGT
TTCGCACCAGGAAGGCAGGACTGCGAGCAAGCATGTGTTCTATATAAACGAGGTAGTGATCTATCTTAGTTTATGTAGGGAATA
GAAACCTACTACATTACCCTTCCAGCACAACGTGCCTATTTGTTCAACGCTGACTCCGTCATCTAGGCAATAACAGTAACAAGG
TCATTATCTTCCTTCACGAGAAGGTAACGTACACACATCTATTTCCCGTCGGGTTCCTAAACAGGTTTTTATTGAGAACATCGGC
GTGCCCCATCAGAGGCTACTACAAGTGAAAATCTATTCTATTCTAGAAATACAATTGAGGATTACTATTTTGTCATCCATCCATTT
TAGAGAATTTCCATCAGATAGTTTGCTATTTTACCACTCGTTGTCGCAAAACCTCGGAAATTCTTAAGGAGCCATTTTTTGGATTA
CTCTCGCGACCATCACCCTATGAAACAACAAGCAACGATGCATAGTGATATATAAACGACTCTTTTTGTAGAAATAGCTTAGGGA
TAAATATATAAATAAAGCCTGCTCTCTTGCTGCGCTTCGAAAGCCACACTTTACAACGCTTGAGGAAAACGGGATAGAAAATATA
ATGGGATATAGCGCCTTCACGTTTTTTGAAGGAAAATACAAGCCAATTGTGACCTTTTCGTTATAAATATAATCTATAAACACACG
CGAATTTTGCAGAATTTTTGAAAAATCGACAAAACATGACAGCCAACTCAATGCTAGATTTCATGTTTGACAAAAGCAGACTCTG
ACTTGTTACGTCGTGTAAGACAGAACAGGTCGGGAAAATTGCTTGAAAGAAACCTTTGCAATTGTAGTAGTGTCATTGCAATAG
AGTATGGAATGCCTTGCTGTCTCTCTGGATAGTTTAACTTCTTAATTGGAGGTCATCTTGTGTAAATGCGACGAGATATTTGTAG
ATTTAGGCGCGCGTAATGTTTATACTGAGGCCAAAAATTGGCGCAAAGCGTATAACCGAAATCTTAAAAAGCCCTTGTTAAACTA
AAAAAAAATAACCCTCACTCAAACACCCTAAAATCATCTTTGAAGACGCATGATTTGCCTTCTTAGATGGTAGATATAAATCGGA
GATTGTTTCACGGTACTTGAAAGAATAGCCCAATTCTCTTGGAACAACCTTTCCGCCTGCGTGTTGTTAAGATCCAGAATTAGC
GCGGCCTGCCCGAGTTATCCAAGCGTGAACTAAAAATAGGAGTACCGTTGGGTAGTAGCGACGCTTCTTTTCATGCTTTACTC
TAGTGGAGGTTGGCCCAACTTATAAGTATAGTCGTATGTTAGGTACAGGCGAAAATATTTAGCCAATCCAAACAAATCTAAAAAA
AAATCCTAGCGAAACATTTCTAGAAGCTTTCTGTCGTTTTTTAATGCTATTATCGTTATTTTGTGCGCTAAAATACACAAACAAAAT
TTTAACGGTAACCCAAGTCGTATTCTTAAATAGGAGGCAAAACAGTAAAATAAAGCTATATAATACTATCCATTTAATAGATTAGGT
CTATTCCAATAATAAATAAAGTATTTAGCCTGTAGAGCAACAATTTTATCAACCTATTTGCTTTTTCCCTCACAAATTTTTGCATTAT
TTCTGGAAGTGCACAACGCCATAAAATAAGGCTCTTTAATTTAATGAAAGCAGACTTTCGAGCTTTATTTTTTTGCAAGTTAACAA
GCCACGAGACAGGTGTTGAAAGATACCGTATCTCTCCGTGGCCTACAGTAAACCCGGCTCACTGTATTAGTCCCAAAGTTTCT
TCTTTCTTTCTATCAGTTCAAGAGAATTACACCTTATTTGTAAAGAGAAAACAAAAAGGACGTTCGAAAACAAAGGAGAGGCGG
CAAGTGTAATGAGGTTTATTTTAGAAAGAAATAGGGCGTCCCTGCGCTAATAAACAGTGAATAATACTACCAATAGCCCGAGGTA
ATTTTCATAACTAGGCCAATCGCTACTTCTAAAGGAAACATTTCATTTAACCTCAGCTAATTTGCCGTCTCAAAACTCGAGTAGC
CCCTGGGTAAGGAAATGAGACTATTTGGCATCTCTTGTTGTAGTGTCAGTTAGTTAGCGCTCAGTGTTTGGTAACGTTTAGAAA
GCGATTATACAAAAGAATAGGGTAAAGTAATCTTCTTTTTACTAAAACACGGTTTAAAAGTAATAAAAATGAAATTGCGGCAATAG
TCAACAAAAGCTTTCTCGTCATCGCACGCATTTTACACGCCGCAAAGAAAGGTCTGAAATTGGTTAAATGGTCCGCCGTAAATT
CGAGCGCGTCATGGTAGACGCGCCTAGAAGTTCAGAGCGAGCTCGCGACTCAACAGTCTTCGCAAAGTTTGCTCTCAAACTA
AGCGCGCAAACATCGTCAATGTATTGCATAAACAAGGAAAATCGCAAACACGGAATGAGTCGTATTTACGAGTTGATCACCGAT
AAATAGGCATGTCAATAGCCCCAGGGGACGCGTCCGATTGCTCACTTGGCGAAATAGTTCGAAAAGGGGTGCACTTAAAAAG
CTCGGGCAAAAAGACAGAACGTTAAAGAATGCATACATGGAGACGCGGACTAATCGCAGTTAAGTCCCGACAACGGAGTCCC
ATAAGATTTTTTTCTCGAGATCCTTGCTTTAGTGATTCCCTGTGTAGTAACAAAATTACCGATATAAGGGTAGTTAGAAACCTTAG
TTATTGCAGGAAGACAATTTCTTACAGCTGTCTGAGAAAACACCTGGATTGGGCTTTTGTGGTATGATTGACGTGAGCGAAATT
TACTAATTAATTGCACTGGCTTCCGGAAATGAGAAAAAGGATTCTAAAAAATTCAGTGTCTGTTCCACTACCCACGCGCGCACG
ATTTGCTGTCATCCCTGATGTTTTTTCTTGCCGCAGAGTTTCCGATGGCAAGTACGCTCTTGGTGCGAGGGTAAACAGCTACAT
GCAACACACACAATAATAGAGACGTCGCAAACAATTATGCACTGAATTACTGACGTGAGAACAATTTGCCATTATTTATTAAACA
AAACCTACGTCGGTACGCAAGTCGCGTGATTTTTTCTATTACCTTTTTGGCTCTAGAAATTCCAGAAAATATTCAAGACGCTATAT
ACGGTTGGCACTAAACCACAAAAGCAGGTGGCGGCCGATTTATGTTATAACTGTTAAGTGAAATGCGGCAGGCAATTTAATACG
TAGTGAACGATTCTCAATCGTCCGGACAATTGAGTTAAAAGCCTACCGAAAGGAAAGAGCTTTCAGGAAAAGAATAAAGCGAG
TTTCCCGACGTAATTGCCATTGTTTGTGCTTGCGCTGTGCAGCGATTGTGATGTAAATGGTTCTGACGGGGCAGTGTAGTGAC
AGGGCCCTGGTTACGGCTAAGGTTCTCAAGAATGCGAAAAGTGCTGCGGAACACTTCGCGCGGGGCAAGCGTGACGTCAGC
CCTTTGATAAATTTGATGCGGCTAAATGAGTTTGCTTTTAGAGATCTGTCCAATGTGAAGTGCGCAGAGAAAGCTATCTATGTCC
TTCCTGAGATACCGAGTTATCATCCCATCTAGACCCGACCATATTGCCTTCACCGGCATAACAAAGTTGTAAGCCCCCGTTTTC
TGTTGACTTTTTGGATTGGAGATAGGACAAAGCTATAAAGAGTCCTATCGAAATAATTATATTTGCTCATGAATTATGAAACGGCA
ATTTGTTTGTAAGCCACTGTGATGGACATGTGATAATGGTTCTAGTAGGATTTAATAAAATGGTTTTCCCTAGCTAGCTACAAAGA
CTCCAGCGCAAACAATTGGCGGGCTCGGGCGAGAGCTTTCCCGGTAAACAATGGAGGGGGAGGAGCTACCGTTTATGTCAC
GGTTAGCTTGTTGACGGACTGTCACTAAAAATAAAACTTCTTTATTACTAGGAGGTAATCTGCACTAGTCGCTAGCGGAGACAC
AGTGAACACCAACACCAGCAGCGCGCAACGTCAACCGGCAAACTGGATCCTCAGTACAGAAACCTCGAGATAGAG

Table S4. Cloned promoter sequences for generation of Betalaminin and FoxA reporter lines
