# Supplementary material for: Notch coordinates self-organization of germ layers and axial polarity in sea anemone gastruloids
Source: Nat Commun. 2026 Jun 19;17:6182. doi: 10.1038/s41467-026-74441-x (PMC13370024; doi:10.1038/s41467-026-74441-x)
Supplement: Supplementary file 3 — Description of Additional Supplementary Files [file 41467_2026_74441_MOESM3_ESM.pdf]

Description of Additional Supplementary Files

File Name: Supplementary Movie 1
Description: Rapid ectodermal epithelialization in developing *Nematostella* gastruloids visualized using **β**-catenin-sfGFP knock in line.

File Name: Supplementary Movie 2
Description: Timelapse imaging showing ingression of peripheral mesodermal clusters visualized by *SnailA::mCherry* (red) with all cells also labeled with SiR-actin (cyan).

File Name: Supplementary Movie 3
Description: (left) Migration and clustering of mesodermal cells (in green) visualized early in *Nematostella* gastruloid development using the ***β**-laminin*::eGFP-CAAX transgenic reporter line. (right) Surface Intensity plot showing intensity maxima representing mesodermal cells clustering.
