# Supplementary material for: Notch coordinates self-organization of germ layers and axial polarity in sea anemone gastruloids
Source: Nat Commun. 2026 Jun 19;17:6182. doi: 10.1038/s41467-026-74441-x (PMC13370024; doi:10.1038/s41467-026-74441-x)
Supplement: Supplementary file 7 — Reporting Summary [file 41467_2026_74441_MOESM7_ESM.pdf]

nature portfolio

Corresponding author(s): Ulrich Technau

Last updated by author(s): May 20, 2026

Reporting Summary

Nature Portfolio wishes to improve the reproducibility of the work that we publish. This form provides structure for consistency and transparency in reporting. For further information on Nature Portfolio policies, see our Editorial Policies and the Editorial Policy Checklist.

Statistics

For all statistical analyses, confirm that the following items are present in the figure legend, table legend, main text, or Methods section.

n/a	Confirmed	
☐	☒	The exact sample size (*n*) for each experimental group/condition, given as a discrete number and unit of measurement
☐	☒	A statement on whether measurements were taken from distinct samples or whether the same sample was measured repeatedly
☒	☐	The statistical test(s) used AND whether they are one- or two-sided<br>*Only common tests should be described solely by name; describe more complex techniques in the Methods section.*
☒	☐	A description of all covariates tested
☐	☒	A description of any assumptions or corrections, such as tests of normality and adjustment for multiple comparisons
☒	☐	A full description of the statistical parameters including central tendency (e.g. means) or other basic estimates (e.g. regression coefficient) AND variation (e.g. standard deviation) or associated estimates of uncertainty (e.g. confidence intervals)
☒	☐	For null hypothesis testing, the test statistic (e.g. *F*, *t*, *r*) with confidence intervals, effect sizes, degrees of freedom and *P* value noted<br>*Give P values as exact values whenever suitable.*
☒	☐	For Bayesian analysis, information on the choice of priors and Markov chain Monte Carlo settings
☒	☐	For hierarchical and complex designs, identification of the appropriate level for tests and full reporting of outcomes
☒	☐	Estimates of effect sizes (e.g. Cohen's *d*, Pearson's *r*), indicating how they were calculated

Our web collection on statistics for biologists contains articles on many of the points above.

Software and code

Policy information about availability of computer code

Data collection	n/a
Data analysis	Nikon NIS Element AR, Leica LAS X, vision Works LS, R-studio

For manuscripts utilizing custom algorithms or software that are central to the research but not yet described in published literature, software must be made available to editors and reviewers. We strongly encourage code deposition in a community repository (e.g. GitHub). See the Nature Portfolio guidelines for submitting code & software for further information.

Data

Policy information about availability of data

All manuscripts must include a data availability statement. This statement should provide the following information, where applicable:

- Accession codes, unique identifiers, or web links for publicly available datasets
- A description of any restrictions on data availability
- For clinical datasets or third party data, please ensure that the statement adheres to our policy

All data are available in the main text or the supplementary materials. Raw sequencing data are deposited at GEO (information given in the data availability statement.)

Research involving human participants, their data, or biological material

Policy information about studies with human participants or human data. See also policy information about sex, gender (identity/presentation), and sexual orientation and race, ethnicity and racism.

Reporting on sex and gender	n/a
Reporting on race, ethnicity, or other socially relevant groupings	n/a
Population characteristics	n/a
Recruitment	n/a
Ethics oversight	n/a

Note that full information on the approval of the study protocol must also be provided in the manuscript.

Field-specific reporting

Please select the one below that is the best fit for your research. If you are not sure, read the appropriate sections before making your selection.

[x] Life sciences [ ] Behavioural & social sciences [ ] Ecological, evolutionary & environmental sciences

For a reference copy of the document with all sections, see nature.com/documents/nr-reporting-summary-flat.pdf

Life sciences study design

All studies must disclose on these points even when the disclosure is negative.

Sample size	All experiments were performed either on large numbers of embryos (>100 per condition and replicate) or in the case of more limited gastruloids at the given numbers (>10).
Data exclusions	No data was excluded
Replication	All experiments were replicated at least 3 times, all replication attempts were successful.
Randomization	n/a
Blinding	n/a

Reporting for specific materials, systems and methods

We require information from authors about some types of materials, experimental systems and methods used in many studies. Here, indicate whether each material, system or method listed is relevant to your study. If you are not sure if a list item applies to your research, read the appropriate section before selecting a response.

Materials & experimental systems

n/a	Involved in the study	
[ ]	[x]	Antibodies
[x]	[ ]	Eukaryotic cell lines
[x]	[ ]	Palaeontology and archaeology
[ ]	[x]	Animals and other organisms
[x]	[ ]	Clinical data
[x]	[ ]	Dual use research of concern
[x]	[ ]	Plants

Methods

n/a	Involved in the study	
[x]	[ ]	ChIP-seq
[x]	[ ]	Flow cytometry
[x]	[ ]	MRI-based neuroimaging

Antibodies

Antibodies used	In this study, rabbit polyclonal anti-GFP (abcam290, RRID:AB_303395) was used. As commercial secondary antibodies, anti-Rabbit IgG AP secondary antibody (Invitrogen; #31346), anti-Digoxigenin-AP Fab fragments (Roche 11093274910) were used.<br>The following custom made antibodies were used: Primary mouse anti-Cdh3 antibody, rat anti-Cdh1, rabbit anti-FoxA and rabbit anti-SnailA.

Validation	see Pukhlyakova EA, Kirillova AO, Kraus YA, Zimmermann B, Technau U. A cadherin switch marks germ layer formation in the diploblastic sea anemone Nematostella vectensis. Development. 2019 Oct 11;146(20): dev174623. doi: 10.1242/dev.174623).

Animals and other research organisms

Policy information about studies involving animals; ARRIVE guidelines recommended for reporting animal research, and Sex and Gender in Research

Laboratory animals	Sea anemone Nematostella vectensis cultured at the Department of Neuroscience and Developmental Biology of the University of Vienna. All embryos derive from the F2 offspring of the cross of the original CH2 and CH6 individuals, isolated by Cadet Hand and published in 1992 (Hand and Uhlinger, 1992). This is stated in the Methods section. This is also the strain that was sequenced as a draft genome (Putnam et al., 2007 and at chromosome scale (Zimmermann et al., 2023). All gastruloids were generated from gastrula stage embryos, as also stated in the Methods and main text.
Wild animals	n/a
Reporting on sex	n/a
Field-collected samples	n/a
Ethics oversight	No ethical approval or guidance was required for working with embryos of this not endangered invertebrate species according to the Austrian national regulations.

Note that full information on the approval of the study protocol must also be provided in the manuscript.

Plants

Seed stocks	n/a
Novel plant genotypes	n/a
Authentication	n/a
