# Supplementary material for: Notch coordinates self-organization of germ layers and axial polarity in sea anemone gastruloids
Source: Nat Commun. 2026 Jun 19;17:6182. doi: 10.1038/s41467-026-74441-x (PMC13370024; doi:10.1038/s41467-026-74441-x)
Supplement: Supplementary file 2 — Peer Review file [file 41467_2026_74441_MOESM2_ESM.pdf]

nature portfolio

Peer Review File

Notch coordinates self-organization of germ layers and axial polarity in sea anemone gastruloids

Corresponding Author: Professor Ulrich Technau

This file contains all reviewer reports in order by version, followed by all author rebuttals in order by version.

Version 0:

Reviewer comments:

Reviewer #1

(Remarks to the Author)
The manuscript by Narayanaswamy et al. uses gastrula reaggregates and embryos of the sea anemone Nematostella vectensis to study cell- and germ layer-intrinsic properties of self-organization during endo-/mesodermal boundary formation and axis induction. The work is based on several publications from the Technau lab where reaggregates were previously used to show that the organiser function relies on Wnt1- and Wnt3-expressing blastopore lips cells to induce the main embryonic axis.

Here, the authors report an impressive technical advancement in generating large numbers of mid-gastrula reaggregates with high consitency and reproducibility in Nematostella. Using high-quality in vivo time imaging combined with new and previously published reporter lines, the authors characterise the reepithalisation of aggregates, and the formation of mesoderm-like clusters at the periphery. Determining the structure, composition and self-organisation of these clusters represents one of the key points of the paper. Using in situ hybridisation and scRNA-Seq, the authors show that these clusters consist of spatially separate endo- and mesoderm identities, whose boundary is dependent on a functioning Notch pathway, and that the endoderm adopts partial mesoderm-like properties, in particular Cadherin1-mediated adhesion properties. Another major outcome of the study is that the induction of polarity in reaggregates by Wnt1 and Wnt3 is dependent on Notch signalling, which together with other data implies a Wnt-Notch feedback loop supporting that process.

In general, the experimental part is well done and solid, and the conclusions well supported by the data. The paper further reinforces the strength of Nematostella reaggregate as model to study fundamental aspects of patterning and axis formation during embryogenesis. Technically, the in vivo timelapse recordings are a highlight of the paper. The work combines a number of state-of-the-art approaches (gastruloids, transgenics, scRNASeq, morpholino knockdowns, chemical perturbations) in a creative and informative way that together support a highly compelling scenario of germ layer and axis reestablishment shown presented in the clear and beautiful Fig.6.
Overall, I think the study is well done and I have only few concerns that would necessitate further experiments. I found however that the manuscript was quite difficult to read, and I have a longer list of minor issues that need clarifications and suggestions that would overall help to make the manuscript easier to read.

I think this manuscript is a strong candidate for Nature Communication and I would highly recommend publication after revision.

Major issues (experimental):
- In l. 100-103 and Fig. 1C, the authors claim that the number of FoxA+/SnailA+ modules is proportional to the seed count. While this experiment looks highly reproducible, it is not possible to claim proportionality based on just those two seed counts. To test proportionality, the authors need to use more seed counts, (f.ex. 20k, 40k, 60k) and a linear regression.
- In l.109-113, the authors present an impressive in vivo imaging movie showing how apical b-catenin reforms in aggregates, supporting the idea that adherens junctions reform during ‘epithelialisation’. As b-catenin localisation could temporally precede the formation of junctions, this video does not fully clarify to what extent epithelia reform in parallel to apical b-catenin relocalisation. This claim would therefore need some more evidence, for example that these cells have adopted an apical-basal polarity (f.ex. by doing z-stacks of the b-cat line) and that apical b-cat localisation indeed coincides with junction formation, f.ex. by showing cadherin co-localisation or by electron microscopy as gold standard.
- L. 136-38: ‘ingression seems only possible where ectodermal epithelization has not yet been completed’: This is a

potentially very intersting observation but am I right to assume that it relies on only a single video? In addition, the video is not very clear to show where epithelization has occurred and where not. Some more experiments, or a more careful wording is necessary here.
- l. 154-157, the authors compare late reaggregation stages with normal planula larvae, claiming that after 48hpa, the body plan is roughly comparable with the postgastrula planula larval. While all the data presented point into this direction, the authors have not shown any evidence that the aboral side, where the apical organ develops, is present in reaggregates at this stage. I think that showing this with a few markers by ISH would be an important addition to the paper as it strengthen this claim and add further evidence that not only the oral but also the aboral pole is induced properly in aggregates.

Major issues (paper structure/clarity)
- The proper understanding of the experimental logics and outcomes requires a lot of previous knowledge about relative expression and interaction between genes (FoxA, Bra, Snail, Notch, Delta, Wnt1, Wnt3, …) specifically at gastrulation in Nematostella. A summary schematics would be very helpful.
- The supplementary figures are difficult to digest (a bit of a mess, really…):
o Why are they numbered 2.1, 2.2, 2.3, 3.1,…? Why start with 2, and why not just numbering them 1,2,3,4…?
o Many figures lack internal letters, f.ex. S3.5, S4.1 or S3.3., which has A and B but 40 panels & inlets.
o The order of Suppl Figures seems random, starting with S4.5B (l. 121), which makes finding the right on a bit like a wild goose chase
o More minor issues below
- I generally found the paper quite difficult to read. In the results part, the different experimental parts may be better connected, with clearer transitions and connections between the parts. The last paragraph (l.288-302) appears particularly unconnected. In the discussion feels, the different parts all seem relevant, but in total the discussion felt a bit too long and could benefit from some shortening and streamlining.
- An aspect connected to the previous point is that in the middle of the manuscript the experimental focus pivots from the aggregates to the embryo (p. 9), almost without any justification.

Minor issues
Please clarify:
- L.28 & other places in the manuscript: The authors need to clarify the meaning of 'endomesodermal'. Is it used in the sense of 'mixed identity' or 'mixed fate'? In bilaterian development, endomesoderm is mostly referred to as 'having both endo- and mesodermal fate', and as I understood the data, the authors rather mean 'endoderm identity with some mesodermal features' here. Also: does it exist in a normal embryos? And is its 'mesodermal' character only defined by the adhesion properties (Cadh1), or does it also express 'mesodermal' transcription factors?
- L.34: I do not understand how 'adoption of ancestral regulatory networks' enables the diversification of body plans. In my understanding, diversification needs novelty, which is the opposite of 'adoption of ancestral'.
- L. 6: why 'tissue(!) symmetry breaking' if in the next sentence it is explained that this occurs often in the egg.
- L.65: it is incorrect to write that 'gastroderm corresponds to mesoderm' as gastroderm also includes septal filament (which derives from endoderm as the authors correctly mention).
- L. 69-70: I do not fully see how the authors have addressed the 'self-organization' of the organizer. They have previously shown that a few (unorganized?) Wnt1-expressing cells are enough to make an organizer, so what do they mean by its 'self-organization?
- FoxA2::mOrange2 line (l. 91) and SnailA::mCherry (l. 129): are those published lines? If yes, they miss citations, and if not, they miss description in the methods.
- Cell numbers in the first results paragraph (70% get incorporated, requirement of ~155 cells): it is unclear to me how those were determined, maybe a few words about the method would help.
- L. 98 & 171: 'staining', 'stained': by what?
- L. 112: 'previously described': reference missing
- L. 118-129: How is the new b-laminin::eGFP-CAAX different from the SnailA::mCherry line (beyond the diff. in fluorophore)? And what is the laminin line showing that the SnailA line does not show? This needs to be addressed in the text.
- L. 141: what is dFISH?
- L. 143-4: 'together with the fact that…': it is unclear what the basis of that 'fact' is' Missing reference?
- L. 145: 'endodermal cell population is also(?) initially migratory post-dissociation': to what refers 'also', does it imply that endoderm is migratory also in embryos? If so, what is the evidence for it?
- L. 150, l. 163: 'foxA+ cells exhibited intercalation': I have a hard time to understand here and throughout the manuscript what 'intercalation' means in this context. Intercalation between what? Or do the authors mean 'mixed with other cells'?
- L. 150-1: This sentence implies that Bra+ cells are endodermal but do not express FoxA+. If so, how does that compare to the gastrula? This needs to be more explicitly explained in the text.
- l.166: 'depolarization of endoderm': what does that mean? Of the cells? Polarity within the foxA+ cluster? Or of the whole gastruloid?
- L. 178: what is a 'cell state' (endodermal) and how is it different from an 'cell identity' (ectodermal and mesodermal; same sentence)?
- L. 179: 'sought to find out if pre-existing endoderm is essential': essential for what?
- L. 186: the 'expanded panel' (Fig. S3.3) shows many more genes than the ones directly described in the manuscript. What about the rest?
- L. 190-16: the description of gene expression is unclear, mainly because the expression patterns are very poorly visible on Fig. S3.3. Also descriptions like 'Delta has an expression maximum at the boundary with the endoderm' are very hard to understand without any additional morphological context.
- L. 206: what is meant by 'endodermal polarity'? I think the use of the term 'polarity' is confusing throughout the manuscript

as it often remains unclear if it refers to the polarity within the tissue (so within endoderm here) or whether the endoderm confers polarity to the rest of the aggregate/embryo.
- L. 212: Is ‘wnt1/3’ a single gene or does it refer to two genes?
- L. 216-18: the statement ‘Notch signalling acts downstream’ is contradicting the model of a ‘feedback loop between Notch and Wnt’ (where no gene would be ‘downstream’ one another, but where both reinforce each other)
- L. 231, 277-9: What analysis was done to determine if genes are ‘up- or downregulated’ based on the scRNASeq datasets? Is that within a cluster, or over the whole dataset? And was that somehow quantified and/or statistically tested?
- L. 238-9: I have no idea what an WGCNA analysis is, and what the authors mean by ‘specific cell type responses to the drug treatment’. What kind of response? And why is one the modules termed ‘pink’? I suggest using a more biologically meaningful terminology.
- L. 268-9: I do not follow the thought that global downregulation of cadh3 by Notch inhibitor is ‘potentially through Notch signalling mediated downregulation of Wnt signalling’.
- L. 271: ‘To further validate this’: what specifically?
- L. 271: ‘cell states’?
- L. 277-9 & Fig. 5B,C: the changes in gene expression are difficult to follow as the figure is too small
- L. 284: ‘we found many genes characteristic of undergoing EMT’: Confusing wording as genes do not undergo EMT. Also: what analysis is this based on? GO term? What does ‘many’ mean? And what are the types of genes (transcription factors, cell adhesion, cell migration)?

Minor issues on Figures and Supplementary figures:
- Many confocal imaging show green/red combinations, which are not colour blind-friendly. Please change.
- Fig. 3C: The legend does not indicate whether a mean or median is plotted. Also a barplot is not appropriate to represent the data as it does not indicate the variation of the datapoints. Please use boxplot.
- Fig. 4: A-C: too small.
- Fig. 5 A, B: too small.
- Fig. 5 C: the overlap and segregation implied in the text is difficult to see. I feel that the merge with DAPI does not add anything, so removing it could make space to increase sizes. Also, a colour choice where overlap would result in white would help identify overlap.
- S2.1: Description of box plots missing. What does n correspond to? Number of clusters or gastruloids?
- Fig. S3.2A: Here in all other figures with quantifications. These are binary, so how were the classes defined…based on what criteria? F.ex. if for Wnt1/40µM, it says: 264/365, how do the 101 embryos look like that did not match the criteria? Did they have stronger staining, or weaker, or were both?
- Fig. S3.2B is missing an x-axis description. Also, use of boxplot is highly recommended.
- Fig.S3.3: Images too small, inlets impossible to see. It was very confusing to see 48h samples positioned left of the 24h sample. Same for the DMSO. Less confusing but still unsual are the gene names on the right border.

Reviewer #2

(Remarks to the Author)
This is an exciting manuscript about the development of Nematostella gastrula embryos from dissociated and reaggregated cells. The manuscript addresses questions of regeneration and de novo pattern formation in the embryonic state. This is a unique conceptual approach because embryonic cells are not fully committed, unlike differentiated cells from adults that are exposed to regeneration signals. The authors refer to these reaggregates as "gastruloids." They specifically studied the processes of germ layer separation and formation of the oral-aboral body axis, both of which are characteristic of this developmental stage. On the cellular level, they analyzed the sorting behavior of cells and found that endo-mesodermal cells sorted out to the aggregate’s surface, where presumptive mesodermal cells form small clusters which had the ability to immigrate into the reaggregate together with peripherally attached endodermal cells. Molecular and functional data indicate that, concurrently with cluster formation, a feedback loop of Notch and Wnt signaling induces polarization of the endodermal cells along the oral-aboral body axis. This ultimately gives rise to a polyp that is structurally identical to those that develop through unperturbed embryogenesis. While these data are new and the experiments are well-designed, some questions remain about how the data are presented and interpreted. These questions should be addressed before publication.

(i) The manuscript focuses on germ layer formation and axial polarity in reaggregates of dissociated embryonic cells. However, throughout the paper description of both topics is not well structured. The paper begins with an Introduction that focuses almost exclusively on self-organization and the molecular signaling pathways involved in the phenomenon of symmetry breaking. Unfortunately, there is no description dealing with the identity of the germ layers in cnidarians compared to bilaterians. This is a topic of intense discussion because traditional textbooks hold that cnidarians are diploblastic organisms with an ectoderm and endoderm, whereas bilaterians are triploblastic, possessing an additional mesoderm. The Technau laboratory has recently addressed this issue publishing several articles that describe the molecular properties of the germ layers. These analyses revealed that anthozoans have a mesoderm. Without an adequate introduction to the molecular homology of germ layers between Cnidaria and Bilateria, readers unfamiliar with this topic will find it difficult to understand the data, particularly in this paper, which describes germ layer sorting. Therefore, it would be helpful to provide an introductory graphical explanation of the germ layer topology of gastrulae and polyps, as well as an explanation of the mesoderm, endo-mesoderm, endoderm, and ectoderm. Figure 1 or 8 in the recent paper “Segregation of endoderm and mesoderm germ layer identities in the diploblast Nematostella vectensis” (doi: 10.1038/s41467-025-63287-4) would be helpful.

Despite the fascinating similarities in the early development of the gastrula stage in sea urchins (deuterostomes) and sea anemones (cnidarians), it would also be helpful to learn why distinct mesodermal and endodermal tissue territories that do

not separate cannot be called endo-mesoderm, as was recently suggested (doi: 10.1038/s41467-025-57109-w). Endo-mesoderm is a characteristic feature of many bilaterian gastrulae before the mesoderm separates from the endoderm.

(ii) The dissociation-reaggregation approach is extremely valuable for testing cellular behavior in scalable systems. The approach—a dFISH analysis of endodermal (FoxA+) and "mesodermal" (SnailA+) cells—convincingly demonstrates organizer formation, which is size-dependent (10,000 cells, one axis; 30,000 cells, two axes). Interestingly, however, there are more clusters than axes, and analyses show that the clusters are much smaller than those that define an axis. This is strikingly similar to the reaggregates in Hydra and raises the question of how inhibition between clusters is maintained. The authors refer to Turing-like mechanisms but should be more precise and also refer to the Gierer/Meinhardt model, which is a nonlinear cross-catalytic model with short-range autocatalytic self-activation and long-range inhibition. Wnt promoter analyses provided evidence for an autocatalytic loop. However, SP5 is a transcription factor that cannot be responsible for long-range inhibition. This is evident in the images the authors present of the size and distances of the Snail A+ clusters. These aspects should be addressed in the Results and Discussion section.

(iii) Figure 2 illustrates germ layer formation using a superfolder transgenic line for β-catenin (sf-GFP). The ingression of "mesodermal" clusters is striking; however, a higher magnification would help demonstrate the corresponding cellular behavior.

(iv) The data on Notch signaling and its role in maintaining endoderm polarity (Fig. 3) are interesting and essential to this paper because they demonstrate that the Notch-Wnt feedback loop can drive endodermal cell polarization. However, the data showing that treatment with the Notch inhibitor LY-411575 leads to the loss of primary axis induction are lacking (see Fig. 3C). Additionally, in Figs. 4A and 4B, the green FoxA signal is nearly indistinguishable from the blue DAPI background.

(v) In general, the readability of the paper could be improved by adding headings that better structure the text, especially in the results section. One option is to create headings based on the headings of the corresponding figures.

Reviewer #3

(Remarks to the Author)
This paper presents a detailed analysis of germ-layer reorganization in Nematostella gastruloids generated by dissociating mid-gastrula embryos and allowing the cells to reaggregate. As a foundation, the authors establish a rigorous and highly reproducible protocol that produces synchronized and scalable gastruloids containing ~155 blastopore lip (Wnt1/Wnt3^{+}) cells—an essential parameter for achieving consistent axis induction. Within 24 hours, these gastruloids reliably reestablish oral–aboral polarity in parallel with germ-layer reorganization, although full germ-layer specification is completed only after 48 hours.

The authors then examine the cellular mechanisms underlying germ-layer formation. Using sfGFP–β-catenin knock-in embryos, they show that ectodermal epithelialization is nearly complete by 24 hours post-aggregation. Mesodermal cells (visualized using SnailA::mCherry) exhibit a distinctive sequence of behaviors: initial externalization toward the periphery of the aggregate, formation of approximately three clusters per gastruloid (each ~20 cells) by 12 hpa, and subsequent ingression of a single cluster to form the inner mesodermal layer. Endoderm formation proceeds more slowly, with epithelialization and inward invagination completed by 48 hpa. Although mesodermal clustering represents a gastruloid-specific feature, the final arrangement of the three germ layers closely parallels that of normal embryogenesis.

The molecular analyses convincingly demonstrate that initial endodermal cells (Wnt1/Wnt3^{+}) are essential for oral–aboral axis reestablishment. The authors further show that Notch signaling is required for setting the mesoderm–endoderm boundary, using both chemical inhibition (LY-411575) and SuH morpholino knockdown. Canonical Notch pathway activity is also necessary to maintain endoderm polarity and identity. Upon Notch inhibition from 24–48 hpf—after gastrulation—endodermal markers such as foxA, brachyury, and Axin are strongly down-regulated in embryos and in gastruloids. scRNA-seq analyses confirm an accompanying loss of endodermal cell types and a reciprocal expansion of mesodermal-like populations, suggesting that Notch activity prevents endomesodermal transition.

Together, these findings support the authors' central conclusion that coordinated interactions between Wnt and Notch signaling couple axial patterning with germ-layer determination in gastruloids. More specifically, they propose a dual activator system in which Wnt/β-catenin drives broad axial patterning while Notch acts as a co-activator that stabilizes endodermal fate.
The discussion of this interaction as an alternative to classical Turing-type models is novel and thought-provoking. Here, Wnt signaling acts as a short-range activator of oral identity, but its activity is spatially constrained by Notch signaling, reducing the need for a long-range inhibitory component as predicted by Turing or Gierer–Meinhardt models.

The proposed link between aggregate size and signaling balance—where aggregates of ~10,000 cells form a single axis, whereas aggregates of ~30,000 cells generate two—is intriguing. Further experimental testing supported by mathematical modeling would strengthen this claim and clarify the underlying molecular logic.

The evolutionary interpretation, however, requires more nuance. The authors argue that patterning mechanisms in cnidarian aggregates have diverged, but comparisons between Nematostella gastruloids and the reaggregation behavior of Hydra or Hydractinia may be confounded by fundamental differences: Nematostella gastruloids derive from embryonic cells, whereas Hydra and Hydractinia aggregates are composed of adult stem cells and differentiated somatic cells. In Hydra, early sorting

of pre-existing germ layers dominates, and in Hydractinia, recent work (Curantz et al.) shows that pluripotent interstitial stem cells drive pattern formation. Direct evolutionary insight would require generating equivalent gastruloids in Hydra or Hydractinia, rather than comparing embryonic-cell aggregates to adult-cell aggregates.

Despite this caveat, the study opens important avenues for understanding the principles that underlie aggregate robustness. Identifying the mechanical, biochemical, and cellular parameters that permit reliable self-organization in Nematostella would represent a major advance and could lead to predictive models applicable across organisms—including vertebrate systems. It would also enable comparative approaches exploring the differences between aggregating embryonic cells and adult stem-cell aggregates across cnidarians.

In sum, this is an excellent and comprehensive study that parallels gastruloid development with embryogenesis while offering new theoretical, signaling, and cellular insights into developmental self-organization. Nonetheless, several points could be refined for clarity:
• A more precise explanation of how axis formation and germ-layer determination proceed in parallel on day one but diverge on day two, as oral–aboral polarity stabilizes while endoderm formation continues.
• Modeling the relationship between aggregate cell number and axis formation to provide predictive value.
• A clearer definition of the temporal and spatial parameters governing Wnt–Notch cross-talk (e.g., number of signaling cells, duration and intensity of signaling, thresholds).
• Finally, an explicit justification for excluding possible contributions of additional signaling pathways.
Overall, this manuscript represents a strong and valuable contribution to our understanding of developmental self-organization in cnidarian systems.

Version 1:

Reviewer comments:

Reviewer #1

(Remarks to the Author)
In their revised manuscript, Narayanaswamy et al. have addressed the major experimental concerns. However, because line numbers are missing from both the manuscript and the point-by-point response, I was unable to fully assess whether all minor issues have been satisfactorily resolved. In addition, the responses are redundantly between 'major experimental', 'major structure' and 'minor issues', which makes it difficult to refer to specific points.

While I went through the authors' replies to both major and minor comments, I was often unable to locate the corresponding revisions in the manuscript, despite the authors indicating that changes had been made. This may again be due to missing line numbers. I therefore recommend that the authors provide a revised manuscript and response letter in which all changes are clearly indicated and cross-referenced to specific locations in the text.

During this check, I also identified several points that would benefit from further clarification.

In some cases, the authors responded to concerns with explanations and references to their previous work, but did not incorporate these clarifications into the manuscript itself. While these explanations are helpful, the authors should not expect readers to be familiar with their earlier publications in such detail. I therefore suggest that all relevant clarifications and references be included directly in the manuscript, not only in the response letter.

A number of minor issues also remain:

Point 3 (major experimental; previously lines 136–138): The response states that “the text has been more nuanced,” but the corresponding sentence appears to have been removed entirely.
Figure 1A: The use of green for activating and similar colors for inhibitory arrows is confusing, as it overlaps with germ layer color coding. These should be revised for clarity.
The authors should follow standard gene nomenclature in Nematostella, where gene names begin with a lowercase letter, in addition to being italic.
Point 2 (minor issues): Although revised, the sentence is now less clear. It is not evident how the “adoption of ancestral regulatory networks” leads to a “diverse morphospace.” This should be reformulated, in particularas this is intended for a general audience.
Point 3 (minor issues; “tissue symmetry breaking”): I thank the authors for their clarification, but I was referring to the 2nd and 3rd sentence of their introduction.The term “tissue symmetry breaking” is not appropriate if the subsequent sentence states that this event occurs prior to or during fertilization, as an egg or zygote is not a tissue.
Point 6: The origin of the foxa2::mOrange transgenic line remains unclear. A foxa2::mOrange2 line was previously reported in Lemaitre et al. (2023; doi:10.1038/s41467-023-39789-4). The authors should clarify whether their line corresponds to this published line or represents a new construct. If the latter, its generation must be described in the Methods.
Point 12 (“together with the fact” sentence): The response does not resolve the issue. The stated “fact” refers to:' together with the fact that mesodermal cells form their clusters by migration and differential adhesion'. Please indicate whether this is shown in the figures or supported by an appropriate reference.
Point 14: This sentence appears unchanged, so it is unclear how the issue has been addressed.
Minor synthax issues remain throughout the manuscript.

Reviewer #2

(Remarks to the Author)
As stated previously, this is an exciting manuscript about the development of Nematostella gastrula embryos from dissociated and reaggregated cells, which presents new and elegant experiments. In my previous review, I raised several points and questions regarding the structure of the manuscript and the interpretation of some experiments, all of which have been carefully addressed.

To clarify the terminology and topology of germ layer formation in Nematostella, the authors have added a new figure (Fig. 1) describing the latest view on germ layer identities, including signalling pathways and associated key factors, as suggested. The cell biological and theoretical aspects of pattern formation in reaggregates have been introduced and discussed from a broader perspective by incorporating classical Hydra experiments and Gierer/Meinhardt modelling. Demonstrating the function of Notch signalling in refining boundaries and adhesion-mediated sorting is an important new perspective. This can also explain classical observations on self-organisation, but provides a deeper molecular understanding.

In addition to these main topics, I also addressed questions regarding experimental, technical and editorial aspects (better imaging of β-catenin in Fig. 2; triple antibody staining of FoxA, cadherin 1 and cadherin 3 in the Notch inhibitor LY-411575 experiments in Fig. 4; improvement of the structure of the paper using headings). These criticisms have also been well addressed.

Reviewer #3

(Remarks to the Author)

The addition of a new Figure 1, in the form of a diagram illustrating the current understanding of the identity of the germ layers (including signaling pathways as well as some of the key transcription factors and adhesion-related genes associated with these layers), clearly helps readers unfamiliar with the Nematostella model system to grasp the significance of this study in terms of development and evolution.

In response to the request to include additional experiments to better predict, through mathematical modeling, how the number of axes that form in the aggregates is determined by the initial number of cells in these aggregates, the authors chose to demonstrate, in a new supplementary figure (Fig. S1), the positive correlation between the number of FoxA+ poles and the initial number of cells in the aggregates. This is an interesting observation, even if it does not investigate the mechanism that triggers, at a certain threshold, the formation of a new axis. I understand that this will be the subject of future work.

I found nothing in the revised version that explains the value of comparing Nematostella gastruloids, formed from embryonic cells, to the reaggregates of Hydra and Hydractinia, produced from adult somatic tissues. Too bad! However, I agree with the authors that Nematostella gastruloids, composed of early embryonic cells, can be considered a useful cnidarian model for in vitro embryonic self-organization, a model that can be compared to vertebrate organoids and gastruloids.

Open Access This Peer Review File is licensed under a Creative Commons Attribution 4.0 International License, which permits use, sharing, adaptation, distribution and reproduction in any medium or format, as long as you give appropriate credit to the original author(s) and the source, provide a link to the Creative Commons license, and indicate if changes were made.
In cases where reviewers are anonymous, credit should be given to 'Anonymous Referee' and the source.
The images or other third party material in this Peer Review File are included in the article's Creative Commons license, unless indicated otherwise in a credit line to the material. If material is not included in the article's Creative Commons license and your intended use is not permitted by statutory regulation or exceeds the permitted use, you will need to obtain permission directly from the copyright holder.
To view a copy of this license, visit https://creativecommons.org/licenses/by/4.0/

Reply to reviewers

Reviewer #1 (Remarks to the Author):
N.B. Reviewer 1 appears to refer to different line and page numbers than the ones of our submitted PDF file. Therefore, in some cases, we were unable to precisely identify the sentence he meant to refer to.. We did our best to find it anyway and hope we could satisfy all the concerns of reviewer 1. Major issues (experimental):

1. In l. 100-103 and Fig. 1C, the authors claim that the number of FoxA+/SnailA+ modules is proportional to the seed count. While this experiment looks highly reproducible, it is not possible to claim proportionality based on just those two seed counts. To test proportionality, the authors need to use more seed counts, (f.ex. 20k, 40k, 60k) and a linear regression.
   Reply: We thank reviewer 1 for this valid point. We carried out additional series of experiments with different cell seed counts and measured the number of oral poles by FoxA+ clusters. The results confirm the correlation of cell numbers and numbers of oral poles. This is now found in Fig. S1.
2. In l.109-113, the authors present an impressive in vivo imaging movie showing how apical b-catenin reforms in aggregates, supporting the idea that adherens junctions reform during 'epithelialisation'. As b-catenin localisation could temporally precede the formation of junctions, this video does not fully clarify to what extent epithelia reform in parallel to apical b-catenin relocalisation. This claim would therefore need some more evidence, for example that these cells have adopted an apical-basal polarity (f.ex. by doing z-stacks of the b-cat line) and that apical b-cat localisation indeed coincides with junction formation, f.ex. by showing cadherin co-localisation or by electron microscopy as gold standard.
   Reply: While this would definitely provide an additional layer of information to the degree of complete epithelialization, we have already followed this in our previous publication (Kirillova et al. 2018) with the use of F-actin stains and confocal imaging where we show clear complete epithelialization by 24hpa, aligning well with our b-catenin knockin line movie.
3. L. 136-38: 'ingression seems only possible where ectodermal epithelization has not yet been completed': This is a potentially very interesting observation but am I right to assume that it relies on only a single video? In addition, the video is not very clear to show where epithelization has occurred and where not. Some more experiments, or a more careful wording is necessary here.
   Reply: The text has been now nuanced to reflect only the likelihood of this scenario.
4. 154-157, the authors compare late reaggregation stages with normal planula larvae, claiming that after 48hpa, the body plan is roughly comparable with the postgastrula planula larval. While all the data presented point into this direction, the authors have not shown any evidence that the aboral side, where the apical organ develops, is present in reaggregates at this stage. I think that showing this with a few markers by ISH would be an important addition to the paper as it strengthen this claim and add further evidence that not only the oral but also the aboral pole is induced properly in aggregates.

Reply: This is certainly an interesting point and we have demonstrated this in our previous work (Kirillova et al. 2018) which includes in aggregates, double colorimetric in situ hybridization stains for *foxA* (oral marker)/*fz5-8* (aboral marker), as well as a single stain for *fgfa1*. However, to address the request of the reviewer, we have now included in this paper, HCR double staining in 48hpa gastruloids, for *brachyury* as a marker of oral identity and *six3-6* as an aboral marker to demonstrate successful reestablishment of aboral identity (Fig. S5).

Major issues (paper structure/clarity)

1. The proper understanding of the experimental logics and outcomes requires a lot of previous knowledge about relative expression and interaction between genes (FoxA, Bra, Snail, Notch, Delta, Wnt1, Wnt3, …) specifically at gastrulation in Nematostella. A summary schematics would be very helpful.
   Reply: We thank reviewer 1 for this point. A summary schematic describing the latest germ layer paradigm, including underlying pathways and markers for the same have been included in the introduction (Fig. 1A,B).
2. The supplementary figures are difficult to digest (a bit of a mess, really…):
   i.Why are they numbered 2.1, 2.2, 2.3, 3.1,…? Why start with 2, and why not just numbering them 1,2,3,4…?
   ii.Many figures lack internal letters, f.ex. S3.5, S4.1 or S3.3., which has A and B but 40 panels & inlets.
   iii.The order of Suppl Figures seems random, starting with S4.5B (l. 121), which makes finding the right on a bit like a wild goose chase
   Reply: As the reviewer suggests, the supplementary figures have now been relabeled to reflect their order of appearance in the text. The figures pointed out by the reviewer have also been changed to include internal letters.

More minor issues below

1. I generally found the paper quite difficult to read. In the results part, the different experimental parts may be better connected, with clearer transitions and connections between the parts. The last paragraph (l.288-302) appears particularly unconnected. In the discussion feels, the different parts all seem relevant, but in total the discussion felt a bit too long and could benefit from some shortening and streamlining.

- An aspect connected to the previous point is that in the middle of the manuscript the experimental focus pivots from the aggregates to the embryo (p. 9), almost without any justification.

Reply:The transition between the result paragraphs have been revised and the discussion has been shortened and streamlined where possible as per the reviewers suggestion.

2.- L.28 & other places in the manuscript: The authors need to clarify the meaning of 'endomesodermal'. Is it used in the sense of 'mixed identity' or 'mixed fate'? In bilaterian development, endomesoderm is mostly referred to as 'having both endo- and mesodermal fate', and as I understood the data, the authors rather mean 'endoderm identity with some mesodermal features' here. Also: does it exist in normal embryos? And is its 'mesodermal' character only defined by the adhesion properties (Cadh1), or does it also express 'mesodermal' transcription factors?

Reply: The reviewer is right that we use the term “endomesodermal” to describe committed endodermal cells that are transiently turning on also a partial mesodermal gene expression. Therefore, to distinguish it from the early embryonic state of endomesodermal fate in vertebrates, we have replaced the term endomesoderm with ‘transient endomesodermal identity’.’

This transient endomesodermal identity is defined by a number of genes shared with mesodermal identity as shown in the scRNAseq data of the gastruloid stages. We only highlight the expression of cadherin1 and the early expression of the mesodermal transcription factor SnailA as key mesodermal markers

- L.34: I do not understand how ‘adoption of ancestral regulatory networks’ enables the diversification of body plans. In my understanding, diversification needs novelty, which is the opposite of ‘adoption of ancestral’.

Reply: We thank the reviewer for this insightful comment. The reviewer is correct in pointing this out and the text has now been revised accordingly.

3. - L. 6: why ‘tissue(!) symmetry breaking’ if in the next sentence it is explained that this occurs often in the egg.

   Reply: Gastruloids can be used to describe how symmetry breaking is reestablished after loss of symmetry among a group of cells. This is particularly useful to understand how axial polarity for instance can be reestablished in these types of in vitro systems. This is a feature difficult to study directly in embryonic systems given that symmetry breaking happens often very early during development (like in the egg due to maternal deposition).

4. - L.65: it is incorrect to write that ‘gastroderm corresponds to mesoderm’ as gastroderm also includes septal filament (which derives from endoderm as the authors correctly mention).

   Reply: The reviewer is right to point this out and it has been modified in the text accordingly.

5. - L. 69-70: I do not fully see how the authors have addressed the ‘self-organization’ of the organizer. They have previously shown that a few (unorganized?) Wnt1-expressing cells are enough to make an organizer, so what do they mean by its self-organization?

   Reply: The term self-organization here refers to how a randomly distributed population of organizer cells are capable due to the inherent properties of the system to undergo symmetry breaking in the gastruloid and reform the future endodermal layer. We have shown that this happens through a Notch signaling mediated mechanism that allows the endodermal cells to “hitchhike” on mesodermal due to their transient endomesodermal features (e.g. sharing the same cadherin1) to reestablish the polarity of the gastruloid with the correct topology of the three germ layer identities along the re-established axis.

6. - FoxA2::mOrange2 line (l. 91) and SnailA::mCherry (l. 129): are those published lines? If yes, they miss citations, and if not, they miss description in the methods.

   Reply: The citation for the SnailA::mCherry line has been added. Cloned Regulatory sequence for the FoxA::mOrange reporter line has been added to supplementary material.

7. - Cell numbers in the first results paragraph (70% get incorporated, requirement of ~155 cells): it is unclear to me how those were determined, maybe a few words about the

method would help.
Reply: As mentioned in the manuscript, we used previous estimates of the number of blastopore lip cells present per embryo determined by fixing, staining the nuclei and quantification from images (Kirillova et al. 2018). Median cells per embryo were determined to be 6934 cells and median number of blastopore lip cells per embryo were determined to be 107 cells. Since we seed 10000 cells per well to reproducibly generate a single axis, we calculated the number of blastopore lip cells seeded per well to be ~155 cells.

8. - L. 98 & 171: ‘staining’, ‘stained’: by what?
Reply: This has been described in the revised text.
9. - L. 112: ‘previously described’: reference missing
Reply: The reference has been added.
10. - L. 118-129: How is the new b-laminin::eGFP-CAAX different from the SnailA::mCherry line (beyond the diff. in fluorophore)? And what is the laminin line showing that the SnailA line does not show? This needs to be addressed in the text.
Reply: Unlike the SnailA line, the □*-Laminin::eGFP-CAAX* line marks the cell boundaries, which we use to assess the epithelial architecture of mesodermal cells. This is especially relevant given our use of it to show the role of Cadherin1 in allowing mesodermal cells to form junctions with each other and, our interest in following how mesodermal cells behave post dissociation during gastruloid development. Additionally, the □-Laminin reporter also expresses a bit earlier than the SnailA reporter.
11. - L. 141: what is dFISH?
Reply: It refers to Double Fluorescent in situ hybridization as we mention in our manuscript.
12. - L. 143-4: ‘together with the fact that…’: it is unclear what the basis of that ‘fact’ is’ Missing reference?
Reply: The fact here refers to the previous statement that endodermal cells (FoxA+) appear to mix with peripheral mesodermal clusters (SnailA+) early during gastruloid development. We have made it clearer in the revised text.
13. - L. 145: ‘endodermal cell population is also(?) initially migratory post-dissociation’: to what refers ‘also’, does it imply that endoderm is migratory also in embryos? If so, what is the evidence for it?
Reply: We refer here to the previous statement describing how mesodermal cells are known to become mesenchymal and migratory post-dissociation in the developing gastruloids. We only highlight that this combined with the fact the endodermal cells are always found to be mixed with these migrating mesodermal cells in our stains, is suggestive that the endoderm is also potentially migratory to some degree post-dissociation. We don't make any claims about the endodermal cells being migratory in the embryo.
14. - L. 150, l. 163: ‘foxA+ cells exhibited intercalation’: I have a hard time to understand here and throughout the manuscript what ‘intercalation’ means in this context. Intercalation between what? Or do the authors mean ‘mixed with other cells’?
Reply: Intercalation here refers to the mixing of FoxA+ cells within the peripheral mesodermal clusters in the gastruloid. We clarified this in the text.

15. - L. 150-1: This sentence implies that Bra+ cells are endodermal but do not express FoxA+. If so, how does that compare to the gastrula? This needs to be more explicitly explained in the text.
    Reply: In the embryo, *brachyury*+ cells overlap with the *foxA*+ domain (determined by RNA hybridization experiments in Lebedeva et al. 2021). However, it extends in a gradient of the oral ectoderm, thus, not all Bra+ cells are also FoxA+.
    - l.166: 'depolarization of endoderm': what does that mean? Of the cells? Polarity within the foxA+ cluster? Or of the whole gastruloid?
    Reply: We apologize for being unclear here. We now changed it to "randomized distribution of FoxA+ endodermal cells in gastruloids".
16. - L. 178: what is a 'cell state' (endodermal) and how is it different from an 'cell identity' (ectodermal and mesodermal; same sentence)?
    Reply: Indeed, these two terms are used as synonyms in the literature. In order to avoid confusion we stick to the term identity in this context throughout the manuscript.
17. - L. 179: 'sought to find out if pre-existing endoderm is essential': essential for what?
    Reply: The reviewer is correct to point this out. We have clarified this now in the revised text.
18. - L. 186: the 'expanded panel' (Fig. S3.3) shows many more genes than the ones directly described in the manuscript. What about the rest?
    Reply: This panel was primarily assembled to identify interesting candidates responsive to notch signaling inhibition for our context of self organization. In the main text, we refer to the previously published SuH (Marlow et al. 2011), since in our hands its expression domain appeared to be the same as that of the Notch receptor, making it an interesting candidate for us.
19. - L. 190-16: the description of gene expression is unclear, mainly because the expression patterns are very poorly visible on Fig. S3.3. Also descriptions like 'Delta has an expression maximum at the boundary with the endoderm' are very hard to understand without any additional morphological context.
    Reply: We agree with the reviewer that the morphological context for the expression maxima of *delta* here is missing, and therefore we have described this further in the text. However, the key point that the broad expression domains of the genes are affected by Notch inhibition are sufficiently clear in our opinion.
20. - L. 206: what is meant by 'endodermal polarity'? I think the use of the term 'polarity' is confusing throughout the manuscript as it often remains unclear if it refers to the polarity within the tissue (so within endoderm here) or whether the endoderm confers polarity to the rest of the aggregate/embryo.
    Reply: We apologize for being unclear on this.In the manuscript, when we refer to 'axial polarity', we refer to the establishment of the oral aboral axis in the entire gastruloid. However, when we mention 'endodermal polarity', we refer to the endodermal cells which are initially uniformly distributed post-dissociation, coming together to form an oral pole. To avoid confusion we now changed the term to 'polarized endoderm distribution'.
21. - L. 212: Is 'wnt1/3' a single gene or does it refer to two genes?
    Reply: Thanks for spotting this.This indeed refers to two genes Wnt1 and Wnt3. The correction has been made in the manuscript.

22. - L. 216-18: the statement ‘Notch signalling acts downstream’ is contradicting the model of a ‘feedback loop between Notch and Wnt’ (where no gene would be ‘downstream’ one another, but where both reinforce each other)
Reply: Although we understand the point of the reviewer, we do not fully disagree with the reviewer on this. In our understanding, a feedback loop between two genes involves both genes/pathways being upstream and downstream of each other. This is in fact, how they reinforce the other’s expression in the case of a positive feedback loop. If we functionally manipulate gene A and see an effect on gene B, it is conceptually downstream.
24. - L. 231, 277-9: What analysis was done to determine if genes are ‘up- or downregulated’ based on the scRNASeq datasets? Is that within a cluster, or over the whole dataset? And was that somehow quantified and/or statistically tested?
Reply: The Reviewer raises an important point. It is true that one cannot make claims of upregulation without providing the necessary DEG analysis between clusters. Since this is technically an observed enrichment of expression based on a combination of the UMAP expression and module scores, we have changed upregulation to observed enrichment.
23. - L. 238-9: I have no idea what an WGCNA analysis is, and what the authors mean by ‘specific cell type responses to the drug treatment’. What kind of response? And why is one the modules termed ‘pink’? I suggest using a more biologically meaningful terminology.
Reply: WGCNA stands for Weighted Gene Co-expression Network Analysis and is a method often used to identify both positively or negatively correlated gene expression sets (also called modules) across conditions or time points in RNAseq datasets. In this case, we identified different gene sets that are correlated as a response to the inhibition of Notch signaling in our scRNAseq datasets. We highlight one such specific module of interest consisting of genes that are normally expressed only in the mesoderm in the control condition, but appear to expand their expression domains to the endoderm in the Notch inhibition condition. This is the ‘pink’ module we refer to. In WGCNA analysis, modules are often described by default as colors. However, as the reviewer mentions, this is not informative for the reader and therefore, we have now referred to this module as the ‘EndoMeso’ module in the manuscript now.
24. - L. 268-9: I do not follow the thought that global downregulation of cadh3 by Notch inhibitor is ‘potentially through Notch signalling mediated downregulation of Wnt signalling’.
Reply: Recent work from our lab (Haillot et al. 2025) has shown that Notch signaling helps maintain Wnt signaling and initiates the high Wnt signaling positive endodermal domain during embryogenesis in Nematostella. Cadherin3 is regulated by Wnt signaling and therefore, we propose that the downregulation of Cadherin3 upon Notch signaling knockdown is indirect due to the downregulation of Wnt signaling, as it extends the range of Notch signaling
25. - L. 271: ‘To further validate this’: what specifically?
Reply: This has now been clarified in the text

26. - L. 271: ‘cell states’?
    Reply: This has now been clarified in the text
27. - L. 277-9 & Fig. 5B,C: the changes in gene expression are difficult to follow as the figure is too small
    Reply: The figure has been resized accordingly
28. - L. 284: ‘we found many genes characteristic of undergoing EMT’: Confusing wording as genes do not undergo EMT. Also: what analysis is this based on? GO term? What does ‘many’ mean? And what are the types of genes (transcription factors, cell adhesion, cell migration)?
    Reply: We apologize for the lack of clarity here. The EndoMeso module consists of mesodermal genes including, but not limited to Cadherin1 and SnailA that are expressed in the invaginating plate known to undergo partial EMT in the embryo. Therefore, we propose two scenarios of which one includes the endoderm displaying features of EMT post dissociation. This behavior of the endoderm however, would need to be confirmed in future by live tracking of endodermal populations during gastruloid development.

<u>Minor issues on Figures and Supplementary figures:</u>

1. - Many confocal imaging show green/red combinations, which are not colour blind-friendly. Please change.
   Reply: we thank the reviewer for spotting this. This has been changed to be color-blind friendly.
2. - Fig. 3C: The legend does not indicate whether a mean or median is plotted. Also a barplot is not appropriate to represent the data as it does not indicate the variation of the datapoints. Please use boxplot.
   Reply: Absolute numbers and their resulting percentages were plotted since the results are binary. There is either axis induction or not and variance wouldn’t be a descriptive metric in this context.
3. - Fig. 4: A-C: too small.
   Reply: Figure has been resized for clarity.
4. - Fig. 5 A, B: too small.
   Reply: Figure has been resized for clarity.
5. - Fig. 5 C: the overlap and segregation implied in the text is difficult to see. I feel that the merge with DAPI does not add anything, so removing it could make space to increase sizes. Also, a colour choice where overlap would result in white would help identify overlap.
   Reply: We thank the reviewer for this useful suggestion. The DAPI field has been removed to make more space for other panels.
6. - S2.1: Description of box plots missing. What does n correspond to? Number of clusters or gastruloids?
   Reply: ‘N’ corresponds to the number of gastruloids used for the quantification. The metrics being measured in ‘B’ are i. No. of clusters per gastruloid and ii. No. of cells per cluster. This is already mentioned beside each graph in the image and additional descriptions are included in the figure legend.
7. - Fig. S3.2A: Here in all other figures with quantifications. These are binary, so how were the classes defined…based on what criteria? F.ex. if for Wnt1/40μM, it says: 264/365,

how do the 101 embryos look like that did not match the criteria? Did they have stronger staining, or weaker, or were both?
Reply: Only staining patterns that were unambiguous were counted for this panel. The rest also appeared to have the correct pattern but were very weak and were therefore excluded in the counts.

8. - Fig. S3.2B is missing an x-axis description. Also, use of boxplot is highly recommended.
   Reply: The axis description has been added.
9. - Fig.S3.3: Images too small, inlets impossible to see. It was very confusing to see 48h samples positioned left of the 24h sample. Same for the DMSO. Less confusing but still unusual are the gene names on the right border.
   Reply: The panel orientation, as well as the gene descriptions have been moved to have a more commonly used representation.

Reviewer #2 (Remarks to the Author):

1. The manuscript focuses on germ layer formation and axial polarity in reaggregates of dissociated embryonic cells. However, throughout the paper description of both topics is not well structured. The paper begins with an Introduction that focuses almost exclusively on self-organization and the molecular signaling pathways involved in the phenomenon of symmetry breaking. Unfortunately, there is no description dealing with the identity of the germ layers in cnidarians compared to bilaterians. This is a topic of intense discussion because traditional textbooks hold that cnidarians are diploblastic organisms with an ectoderm and endoderm, whereas bilaterians are triploblastic, possessing an additional mesoderm. The Technau laboratory has recently addressed this issue publishing several articles that describe the molecular properties of the germ layers. These analyses revealed that anthozoans have a mesoderm. Without an adequate introduction to the molecular homology of germ layers between Cnidaria and Bilateria, readers unfamiliar with this topic will find it difficult to understand the data, particularly in this paper, which describes germ layer sorting. Therefore, it would be helpful to provide an introductory graphical explanation of the germ layer topology of gastrulae and polyps, as well as an explanation of the mesoderm, endo-mesoderm, endoderm, and ectoderm. Figure 1 or 8 in the recent paper “Segregation of endoderm and mesoderm germ layer identities in the diploblast Nematostella vectensis” (doi: 10.1038/s41467-025-63287-4) would be helpful.

   Despite the fascinating similarities in the early development of the gastrula stage in sea urchins (deuterostomes) and sea anemones (cnidarians), it would also be helpful to learn why distinct mesodermal and endodermal tissue territories that do not separate cannot be called endo-mesoderm, as was recently suggested (doi: 10.1038/s41467-025-57109-w). Endo-mesoderm is a characteristic feature of many bilaterian gastrulae before the mesoderm separates from the endoderm.

Reply: We follow the suggestion of the reviewer and add as new Fig. 1 a scheme describing the latest view on germ layer identities (including signaling pathways and some of the key

transcription factors and adhesion related genes associated with these germ layers).
The reviewer is right in pointing out that the term endomesoderm has found primary usage in the literature on bilaterian embryogenesis to describe a group of bipotent cells capable of giving rise to both endoderm and mesoderm. In our case, we describe cells that are committed to the endodermal fate transiently (!) in addition turn on mesodermal gene expression. Therefore, we have replaced the term endomesoderm with 'transient endomesodermal identity' as they return to the endodermal-only identity in the course of development. We think that this is triggered by the loss of Notch signaling as a consequence of the dissociation and hence loss of cell-cell contacts.

2. The dissociation-reaggregation approach is extremely valuable for testing cellular behavior in scalable systems. The approach—a dFISH analysis of endodermal (FoxA+) and "mesodermal" (SnailA+) cells—convincingly demonstrates organizer formation, which is size-dependent (10,000 cells, one axis; 30,000 cells, two axes). Interestingly, however, there are more clusters than axes, and analyses show that the clusters are much smaller than those that define an axis. This is strikingly similar to the reaggregates in Hydra and raises the question of how inhibition between clusters is maintained. The authors refer to Turing-like mechanisms but should be more precise and also refer to the Gierer/Meinhardt model, which is a nonlinear cross-catalytic model with short-range autocatalytic self-activation and long-range inhibition. Wnt promoter analyses provided evidence for an autocatalytic loop. However, SP5 is a transcription factor that cannot be responsible for long-range inhibition. This is evident in the images the authors present of the size and distances of the Snail A+ clusters. These aspects should be addressed in the Results and Discussion section.

Reply: We fully agree with the reviewer on these points. We allude to the Turing model as one often used to describe the emergence of patterns we observe in our system. In the revision, we have now also mentioned the Gierer-Meinhardt model as an biologically even more plausible variant of the Turing-like reaction-diffusion model (which Gierer and Meinhardt invented independently, i.e. not being aware of the Turing model). However, based on our findings, we propose that a combination of global axial patterning by Wnt signaling (driven by a Wnt-Notch feedback loop), Notch signaling to refine boundaries and adhesion mediated sorting can explain the observations even better and are also able to self-organize. As for the presence of more initial FoxA+ clusters compared to the final oral poles in the polyp, we have briefly mentioned that at least in the gastruloids, the primary FoxA+ poles that go on to determine the axis could be determined through previously described mechanisms such as cell competition (Krammer et al. 2024).

3. Figure 2 illustrates germ layer formation using a superfolder transgenic line for β-catenin (sf-GFP). The ingression of "mesodermal" clusters is striking; however, a higher magnification would help demonstrate the corresponding cellular behavior.

Reply: The focus of this paper is to demonstrate the overall behavior of the germ layers in the context of gastruloid development. We have already tracked with higher magnification, individual mesodermal cells and their ingression in our previous work (Kirillova et al. 2018). We

agree with the reviewer however, that higher magnification imaging for all germ layers in future, would provide valuable additional insights about cell sorting dynamics. At present it is technically not possible during early stages of development. We are imaging the aggregates under the Viventis light sheet microscope, in the wells where they were reaggregated as they are at this point too fragile to be transferred to other microscopes allowing higher magnification.

4. The data on Notch signaling and its role in maintaining endoderm polarity (Fig. 3) are interesting and essential to this paper because they demonstrate that the Notch-Wnt feedback loop can drive endodermal cell polarization. However, the data showing that treatment with the Notch inhibitor LY-411575 leads to the loss of primary axis induction are lacking (see Fig. 3C). Additionally, in Figs. 4A and 4B, the green FoxA signal is nearly indistinguishable from the blue DAPI background.

Reply: It is unclear what is referred to as lacking in this experiment (Fig. 3C, now Fig. 4C). We demonstrate that the previously reported axis induction capacity of Wnt1 and Wnt3 expression (Kirillova et al. 2018) is not sufficient on its own without the presence of Notch signaling to induce an oral aboral axis in the gastruloids.
If the reviewer is referring to the Fig 4D (now Fig. 5) with the triple antibody staining of FoxA, Cadherin1 and Cadherin3, FoxA protein as a transcription factor is expected to localize to the nucleus, as we show clearly in the image. All FoxA signals (in green) must indeed overlap with nuclear DAPI signals (in blue).

5. In general, the readability of the paper could be improved by adding headings that better structure the text, especially in the results section. One option is to create headings based on the headings of the corresponding figures.

Reply. We thank the reviewer for this suggestion. Headers have been added to the section.

Reviewer #3 (Remarks to the Author):

1. A more precise explanation of how axis formation and germ-layer determination proceed in parallel on day one but diverge on day two, as oral–aboral polarity stabilizes while endoderm formation continues.

Reply: Based on the evidence we have so far, we have put forth a model of spatiotemporal progression of both axial polarity and germ layer topology reestablishment, from dissociation to body plan reestablishment in **Fig 7.** of our revised paper. This has also further been elucidated in our discussion.

2. Modeling the relationship between aggregate cell number and axis formation to provide predictive value.

Reply: Instead of modelling, we now provide in the new Fig. S1 new data on how the number of axes (as determined by localized FoxA clusters) scales with the number of cells in the aggregate.

3. A clearer definition of the temporal and spatial parameters governing Wnt–Notch cross-talk (e.g., number of signaling cells, duration and intensity of signaling, thresholds).

Reply: The reviewer touches on a couple of important and valid questions, which are certainly worth investigating in separate subsequent papers. We have, at least in part, estimated the number of organizer blastopore lip cells (Wnt1/Wnt3+) required for the robust generation of gastruloids in the paper. Describing the precise duration and levels of signaling needed to establish the various identities are indeed very interesting as the reviewer suggests. However, to answer this additional and more refined imaging modalities would need to be developed and will certainly be a direction of interest for us in future.

4. Finally, an explicit justification for excluding possible contributions of additional signaling pathways.

Reply: As a juxtracrine signaling pathway, Notch signaling was of particular interest to us in this context of self organization, and how the loss of such close cell contacts post-dissociation could influence tissue identity. Therefore, Notch signaling was the focus of this paper. We have however also highlighted the potential role of other pathways (such as BMP signaling) in gastruloid polarity reestablishment, both in this paper as well as in our previous work (Kirillova et al. 2018).
